# Polymer based advanced recipes for imidazoles: a review

**DOI:** 10.55730/1300-0527.3357

**Published:** 2022-01-31

**Authors:** Shivnath R. PATEL, Rajendra V. PATIL, Jagdish U. CHAVAN, Anil G. BELDAR

**Affiliations:** Department of Chemistry, P.S.G.V.P. M’s SIP Arts, GBP Science and STKVS Commerce College, Nandurbar, India

**Keywords:** Imidazole, polymer supports, nanocatalysts, hybrid catalysts

## Abstract

Imidazoles and their scaffold are an extraordinarily essential class of nitrogen bearing azole heterocyclic compounds. They have different place in wide area of organic synthesis, which can be utilized in a variety of applications in diverse fields including agriculture, medicine, polymer and various industries. Numerous methods for synthesis of imidazole derivatives are reported in last few decades. Existing conventional methods are more or less significant and confined due to its time-consuming reactions, high cost of catalyst, extensive methodologies, low yield rate or no reusability respectively. Overcoming to inefficient conventional methods, finding of novel and effective methods of imidazole synthesis becomes a crucial step in expanding dynamics of material chemistry. The synthesis and design of imidazole derivatives employing polymer-supported techniques will be discussed in this review. In addition, the utilization of polymer-supported organic, inorganic, hybrid, bio, and nanocatalysts in the synthesis process will be discussed.

## 1. Introduction

**Figure 1:**
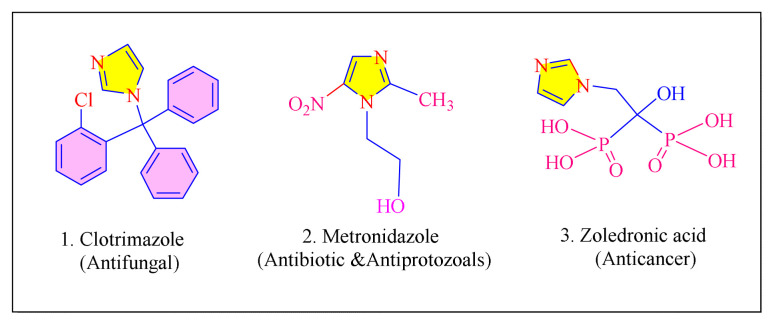
Commercial names and chemical structures of some imidazole cores drugs.

Heterocyclic class containing compounds like imidazole is a great interested in the synthesis of its derivatives. Synthetic and medicinal chemists have been interested in developing novel derivatives for a long time. Because of its ability to treat a variety of unpleasant and awful diseases, imidazole is an important area of pharmaceutical study. It has antibacterial, antifungal, antiviral, antitubercular, antipyretic, antioxidant, antiprotozoal, antiallergic, antiprion, psychotropic, insecticidal, and analgesic properties, among others [1]. It is also found in pilocarpine alkaloids, histidine, histamine, vitamin B_12_, enzymes, nucleic acid bases, and biotin, among many other ionic liquids and natural products [2]. Tealeaves and coffee beans contain a fused imidazole ring, such as the theophylline molecule, which activates the central nervous system. They are commonly present in a variety of synthetic pharmaceuticals, including commonly prescribed medicines includes clotrimazole, metronidazoles, and zoledronic acid (Figure 1) [1,3].

Imidazole compounds have found several industrial applications in agrochemicals including prochloraz, enilconazole, bifonazole, etc. They have also used as functional materials in organic electroluminescent devices (OLED), metalloenzymes, ionic liquids, conjugated and functional polymers, coordination chemicals as essential ligands [4,5]. A new multicomponent salt of imidazole like TBTA-IM (tetrabromoterephalic acid with imidazole) shows optical, thermal and electrical transport properties. Imidazolium chloride or nitrate salts are used as ionic liquids and precursors to stable carbenes [6]. There are varieties of therapeutic and industrial applications for this imidazole compounds. The creation of novel imidazole compounds has piqued the interest of scientists.

Multicomponent reactions (MCRs) play an important role in organic synthesis and are powerful tool for generating molecular complexity. Several conventional methods of synthesis of imidazole and their derivatives have reported but these have some limitations. Because of the harsh reaction conditions, low yield, and tedious work-up processes, as well as the cooccurrence of various side reactions, it is desirable to build a cost-effective, safe, and eco-friendly reaction system. Recently, polymer supported multicomponent reaction has received considerable importance in synthetic chemistry because of the reactions are often very clean and high yielding, work up involves simple filtration and evaporation of solvent, reusability of catalyst, gather selectivity, enhanced reaction rate, low costing and eco-friendly reaction system [7].

Some reviews based on role of polymers as catalyst and catalyst to provide heterogeneous catalysis for different heterocyclic compounds were notable and helps to enlighten the impressive features of polymeric catalysts such as ease of use, increased reaction rates, higher selectivity, easy set-up, and catalysts recyclability. These involve using a polymeric resin or other polymeric solid material to support substrate, which can then be elaborated using an excess of reagent and desired molecule is than detached from the support material and isolated by simple filtration [8–10].

The possible approach of the synthesis of imidazole derivatives is the focus of this review article and design of imidazole derivatives using polymer or solids-supported strategies. The aim of this review is to compile all the literature regarding the role of polymers for the improvements in the synthesis of imidazole. The advancements to the synthesis of imidazoles include reagents supported to the polymers as well as polymers as catalyst and catalyst supports. Organic polymers, inorganic polymers, hybrid polymers, biopolymers as biocatalysts, and are all explained in their own section. Furthermore, nanocatalysts as nanoparticles and nanocomposites that serve as nanopolymers are also covered in this review.

### 1.1 Synthetic development of imidazole moieties

Imidazole as well as its derivatives can be prepared using a variety of traditional methods, including Debus synthesis, Radziszewski synthesis, imidazoline dehydrogenation, imidazole from alpha halo ketones, Wallach synthesis from aminonitrile and aldehyde, and Marckwald synthesis, among others. In general, these methods entail extreme conditions, a variety of name reactions, multicomponent and multistep reactions, Lewis base and Lewis acid, metal-free condition, expensive transition metal catalyst, or in solvent and solvent-free situation. Substituted imidazoles are generally synthesized by multicomponent condensation of either 1,2- diketones, α-hydroxy-, acetoxy-, silyloxyketones, α-ketomonooximes or nitriles with aldehydes and/or primary amines, usually under microwave-assisted and traditional heating, or in refluxing in acetic acid, and/or in the presence of different catalysts. Heinrich Debus first synthesized imidazole in 1858. They employed this procedure to make C-substituted imidazoles, despite the very low yields. Table 1 shows some traditional techniques for the synthesis of imidazoles.

**Scheme 1:**
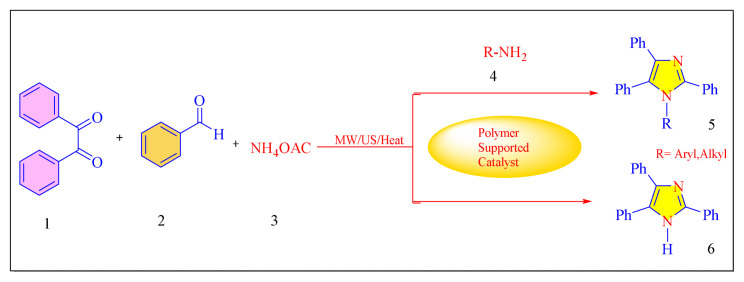
Synthesis of tri/tetrasubstituted imidazoles derivatives.

A number of methods reported to solid or polymer-supported heterogeneous catalysts-assisted synthesis of multisubstituted imidazoles. Multicomponent procedures using the condensation of aldehydes, 1, 2-diketone, and ammonia as the nitrogen source have been used to make various imidazoles. In order to attain superior selectivity and conversion rates, the activities of a wide range of heterogeneous catalysts have been thoroughly explored. In generally, trisubstituted and tetrasubstituted imidazole derivatives were synthesized employing a one-pot cyclo-condensation of benzil, benzaldehyde, ammonium acetate, and primary amines under microwave irradiation and conventional heating with solid or polymer-supported heterogeneous catalysts (Scheme 1) [11].

## 2. Polymer supported inorganic catalysts in imidazole synthesis

In 2020, Subba et al. reported a green and sustainable one-pot multicomponent reactions (MCRs) protocol for 2,4,5-triaryl and 1,2,4,5-tetraryl imidazole compounds by employing a titanium based solid silica-supported catalyst [ TiCl_3_-SiO_2_, Table 2, Entry 1] in the absence of a solvent. This method has various advantages over earlier reported methods; including recyclable solid-supported catalysts, low-cost reagents, nontoxic metal contamination, avoidance of moisture-sensitive metal salts, high yields, a simple work-up process, and a solvent-free condition. Up to the fifth reaction run, the TiCl_3_-silica solid catalyst was recycled and reused with no loss of catalytic activity [12].

In 2015, Gupta and Gupta employed copper (I) oxide supported on silica [SiO_2_-Cu_2_O, Table 2, Entry 2] as a heterogeneous solid supported catalyst for the synthesis of 2,4,5-triaryl imidazoles using ethanol as the solvent. Good to high amount of yield of substituted imidazoles they have reported. Variety of solid supports were screened for the optimization of catalytic activity such as SiO_2_–Cu_2_O, hydroxyapatite–Cu_2_O, basic Al_2_O_3_–Cu_2_O and cellulose–Cu_2_O; among them SiO_2_–Cu_2_O was found to be the most suitable one. The recovered catalyst was reused six times in a row with no noticeable decrease in activity. This approach has several advantages, including simplicity of the use, low cost, and quick response times. Despite the fact that the process appears to be standard, with lengthier reaction times for catalyst preparation [13].

**Figure 2:**
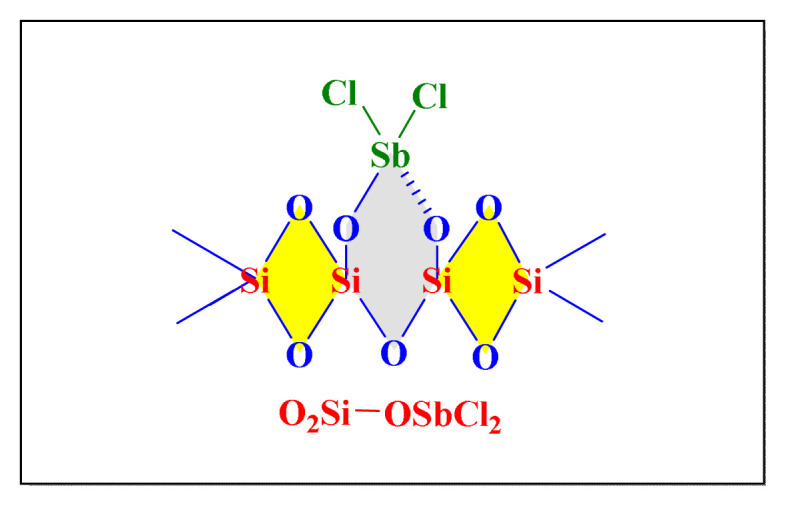
Silica-supported SbCl_3_ catalyst.

Safari in 2014 used antimony trichloride supported by silica [SbCl_3_-SiO_2_, Figure 2, Table 2, Entry 3] as a heterogeneous catalyst to achieve the solvent-free synthesis of 2,4,5-trisubtituted imidazole and 1,2,4,5-tetrasubtituted imidazole derivatives under microwave irradiation at temperatures. With SbCl_3_-SiO_2_ catalyst, the methodology revealed good to outstanding yields and a short reaction time. As a result, using SbCl_3_ dispersed on dry silica gel not only provides good yield, but it also eliminates issues such as catalyst cost, handling, safety, and pollution. This green, environmentally friendly catalyst is a heterogeneous acid that may be easily removed from the reaction mixture using simple filtration. The recovered catalyst was used in model reaction and it is found that after five cycles; there was no notable reduction of activity [14].

In 2013, Chavan et al. attempted conventional solvent-free synthesis of 2,4,5-trisubtituted and 1,2,4,5-tetrasubtituted imidazoles using silica-supported chloride [SiO_2_-Cl, Table 2, Entry 4] as a catalyst. This methodology has a number of appealing advantages, including simplicity of the use, economical availability, and fast response time. Additionally, studied catalysts were reused and recycled for three successive runs, showing no significant loss in activity but seems slightly less impressive due to average to good yields [15].

Rostamnia and Zabardasti demonstrated on the utilization of mesoporous silica SBA-15 and 2,2,2-Trifloroethanol [SBA-15/TFE Table 2, Entry 5] as a metal-free heterogeneous catalyst for the synthesis of trisubstituted and tetrasubstituted imidazoles in 2012. Their procedure has the advantages of being performed in neutral conditions and producing desirable products with higher yields. Recycling as well as performance of SBA-15/TFE investigated and show that catalyst can be used five times at least without a significant decrease in the catalytic activity [16].

Maleki et al. in 2012 have mentioned green method without the use of solvents in order to synthesise trisubstituted imidazole derivatives under conventional heating, by condensation of benzil or benzoin with different benzaldehydes and NH_4_OAc utilizing sulphuric (H_2_SO_4_) acid supported over SiO_2_ [H_2_SO_4_-SiO_2_, Table 2, Entry 6] as a heterogeneous catalyst. This technology offers a number of benefits, including simplicity of use, better yields, and nonchromatographic approaches for product. In addition, the reusability and recycling effect of the catalyst was also tested. It was used three times in a row with no notable decrease in efficiency [17].

In 2012, Kumar et al. demonstrated a three component reaction of 1–2 diketone, benzaldehyde, and acetic acid ammonium salt to form 2,4,5-trisubstituted imidazole and four component reaction involving 1,2-diketone, benzaldehyde, primary amine and acetic acid ammonium salt to form 1,2,4,5-tetrasubstituted imidazole by using a different fluoroboric acid derived catalyst system. Under conventional heating and neat conditions, fluoroboric acid immobilized on chromatographic silica gel [HBF_4_-SiO_2_, Table 2, Entry 7] produced excellent results. It has successfully added a new form of catalyst system to the improvement in the synthesis of imidazoles. The advantages of the presented method have been emphasized by the solvent-free situation, fast reaction time, and outstanding yields [18].

Shaterian et al. in 2011 was employed phosphorus pentoxide supported on silica gel [P_2_O_5_-SiO_2_, Table 2, Entry 8] as a highly efficient and reusable heterogeneous catalyst for the synthesis of 2,4,5-trisubstituted and 1,2,4,5-tetrasubstituted imidazoles. Different active aldehyde and amine compounds were used to prepare substituted imidazoles. Excellent yields, a cleaner reaction, simplified experimental and operation step, and the ability to readily remove the catalyst from the reaction mixture are all the advantages of this innovative approach. The catalyst was also recovered and reused six times without losing its activity [19].

In 2011, Safari et al. have presented a novel silica-supported titanium tetrachloride [TiCl_4_-SiO_2_, Table 2, Entry 9] as a heterogeneous catalyst for the solvent-free synthesis of trisubstituted imidazoles. The syntheses were performed under conventional as well as microwave irradiated conditions. The reaction time was shortened from 60 min to 10 min by microwave irradiation. Furthermore, the yields achieved were good with conventional heating and outstanding with microwave irradiation. The methodology found added a new form of silica-supported catalyst but seems slightly less impressive due to unexplained of reusability of catalyst [20].

**Figure 3:**
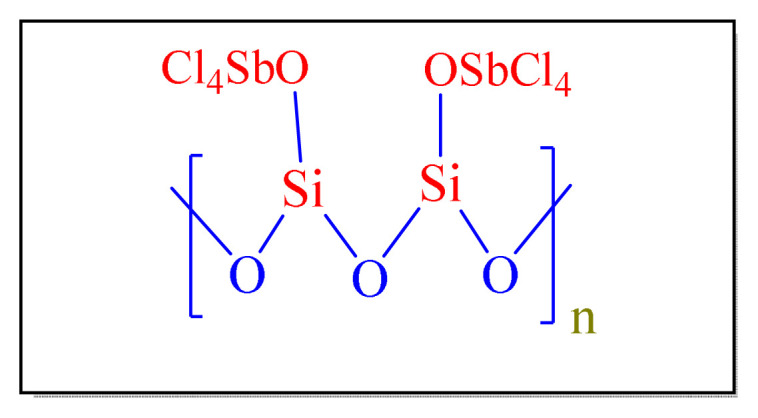
Proposed structure for SbCl_5_-SiO_2_ catalyst.

Sadeghia et al. in 2010 have applied silica-supported antimony pentachloride [SbCl_5_-SiO_2_, Figure 3, Table 2, Entry 10] as catalyst for synthesis of tetrasubstituted imidazoles. According to the obtained data, the adoption of the 62% SbCl_5_-SiO_2_ (10 mol%) under refluxing ethanol or solvent-free conditions seems to give best yields. The catalyst can be removed easily by simple filtration. However, the reusability of the catalyst was investigated in this method by utilizing it three times in a succession, and a significant decrease in its efficiency was observed [21].

**Figure 4:**
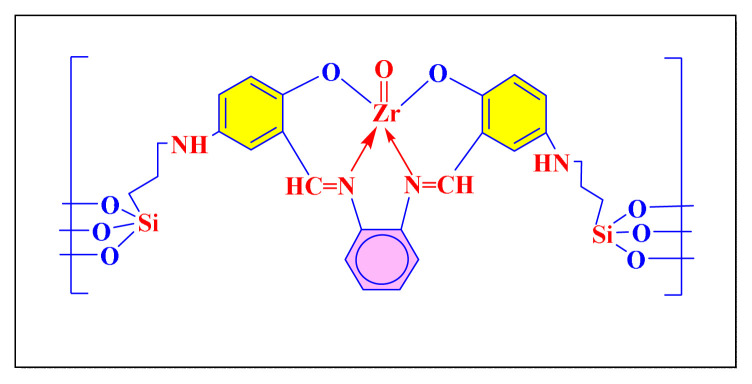
Zr-CAP-SG catalyst.

In 2011, Sharma et al. validated one-pot condensation of various aldehydes with 1,2-diketone and ammonium salt of acetic acid in acetonitrile at room temperature using a zirconium grafted onto the CAP-functionalized silica gel [Zr-CAP-SG, Figure 4, Table 2, Entry 11] as an effective and reusable catalyst. The methodology seems slightly less impressive due to average to good yields and preparation of catalyst requires longer time. Mild reaction conditions, fast reaction times, high TON (Turnover number), a simple work-up technique, and the use of an efficient and recyclable catalyst are all key features of this protocol [22].

**Figure 5:**
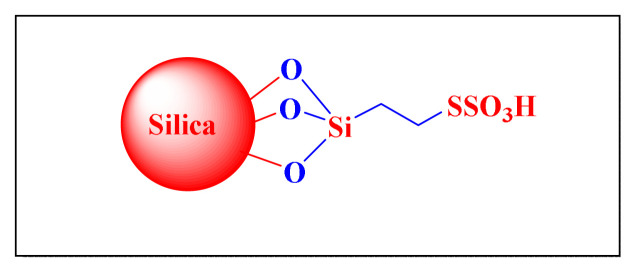
Silica-bonded S-sulfonic (SBSSA) catalyst.

In 2009, Niknam et al. described the synthesis of trisubstituted imidazoles in the presence of silica-supported over S-sulfonic acid [SBSSA, Figure 5, Table 2, Entry 12] as a catalyst at 130 °C under neat condition. The catalytic activity was tested with a model reaction in the presence of varied catalytic amounts of SBSSA under neat conditions, yielding a high yield of 0.002 g (0.068 mol%) of SBSSA at 130 °C. The suggested approach has the following notable advantages: simple reaction set-up and easy separation of the catalyst from the products using filtration. The recycled catalyst could be reused three times without any further treatment. It was mentioned that the catalyst could be reused for three cycles without losing significant activity [23].

**Scheme 2:**
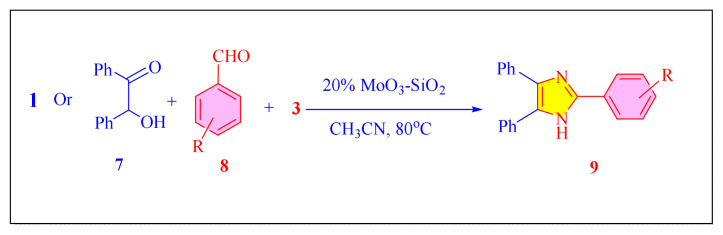
Synthesis of 2,4,5-trisubtituted imidazole compound.

Bhosale et al. in 2009 have developed mild, effective and reusable MoO_3_-SiO_2_ catalyst [Table 2, Entry 13] for the synthesis of 2,4,5-trisubstituted imidazoles using a one-pot cyclocondensation of α-β diketone or α-hydroxy ketone with aromatic aldehydes and NH_4_OAc in CH_3_CN as the solvent, can give reaction yields up to 95% imidazoles [Scheme 2]. The most visible advantage of the MoO_3_-SiO_2_ solid acid catalyst in this methodology is the created ecologically safe approach, clean reactions with no side product, and reusability of the catalyst efficiently for three cycles without any appreciable yield loss [24].

Sadeghi et al. in 2008 have investigated the synthesis of 1,2,4,5-tetrasubstituted imidazoles under heat and solvent-free circumstances. In this approach they used boron trifluoride supported over silica [37% BF_3_-SiO_2_, Table 2, Entry 14] as an efficient, easily available, and recyclable solid acid catalyst. This one-pot multicomponent reaction is relatively simple, yielding good to exceptional yields, and is a table reagent that is simple to operate with easier access of the reactants to the active sites, which makes a vital contribution. The recovered catalyst could be reused for three additional cycles with only a modest loss of activities [25].

Kantevari et al. reported a one-pot, four-component synthesis of 1,2,4,5-tetrasubstituted imidazoles in 2006. They used perchloric acid adsorbed on silica gel [HClO_4_-SiO_2_, Table 2, Entry 15] as catalyst to condense various aldehydes, benzil, aliphatic or aromatic primary amines, and ammonium acetate under thermal and neat conditions. The varied work-up approach resulted in good to exceptional levels yields. HClO_4_–SiO_2_ catalyst also has notable advantages, such as being nontoxic, simple to manufacture, affordable, and readily available, as well as having outstanding catalytic activity. The reusability of catalysts is not addressed in this methodology, which is a significant disadvantage [26].

**Scheme 3:**
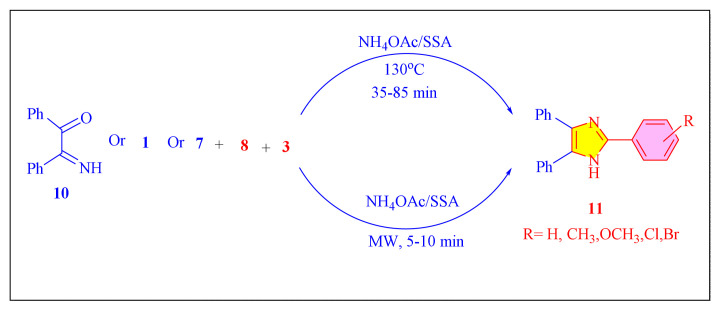
Synthesis of 2,4,5-trisubtituted imidazoles derivatives.

In 2006, Shaabani et al. presented silica sulphuric acid [H_2_SO_4_- SiO_2_, Table 2, Entry 16] as a low-cost acid catalyst for the synthesis of 2,4,5-trisubstituted imidazoles. In this environmentally responsible green approach, simple one-pot cyclocondensation of 1–2 diketone or α-hydroxyketone or α-keto-oxime with different aromatic aldehydes, ammonium acetate. The methodology employed both solvent-free microwave irradiation as well as conventional heating conditions [Scheme 3]. Although the technology produced well to exceptional yields, fewer wastes, simplicity of separation and recovery, and the substitution of liquid acids with solid acids are all desired features for the chemical industry that we took into account in our green chemistry approach. Furthermore, the potential of silica sulphuric acid catalysts to be recyclable reaction medium and reused for four successive reactions negligible drop catalyst activity [27].

In 2006, the group of Hossein have described green chemistry related approach for the synthesis of trisubstituted imidazoles by simple one-pot cyclocondensation of 1,2-diketone or α-hydroxyketone with different aromatic aldehydes, ammonium salt of acetic acid in the presence of sodium hydrogen sulfate supported over silica gel [NaHSO_4_-SiO_2_, Table 2, Entry 17] under microwave irradiation and neat condition. This process produces high-yielding compounds with a fast reaction time and easy set-up, while avoiding the concerns associated with catalyst expense, maintenance, toxicity, and pollutants. This catalyst is nonvolatile, renewable, nonexclusive, easy to handle, and thermally resistant, making it ideal for a range of organic conversions [28].

Balalaie et al. reported an environmentally acceptable method for the one-pot synthesis of 1,2,4,5-tetrasubstituted imidazole derivatives utilizing zeolite HY and SiO_2_ gel [Table 2, Entry 18] catalysts under solvent-free and microwave irradiation in 2000. The yields employing zeolite HY was less than those with silica gel in this comparative investigation of tetrasubstituted imidazole synthesis. In this present has protocol, various key advantages such as reusable solid catalysts are employed, maximum yield, quick reaction times, and easy set-up and work-up. Another notable drawback of the zeolite HY catalyst, which is made from zeolite NH_4_Y subsequently heated for 5 h at 600 °C [29].

Tanna et al. in 2021 have introduced a green system and efficient recovery of the activated fuller’s earth as a natural source catalyst that mostly composed of aluminium magnesium silicate [Table 3, Entry 1] for the synthesis of 2,4,5-triphenyl imidazoles with one-pot multicomponent condensation reaction under solvent-free condition at 100 °C. This technology is a valuable contribution to existing processes because of its good yields of product, quicker reaction time (2–5 h), readily available and inexpensive catalyst, simple experimental and isolation procedure, and environmentally acceptable reaction conditions. The recyclability of the catalyst is found to remain stable across three cycles, with no detectable activity loss [30].

In 2021, Sudarshan et al. achieved environmentally benign approach for the one-pot multicomponent synthesis of 2,4,5-triaryl imidazole compounds by employing ZSM-11 as efficient heterogeneous catalyst [ZSM-11, Table 3, Entry 2] under solvent-free environments through conventional heating. When compared to the previous methods, this approach offers numerous evident advantages, including the avoidance of hazardous catalyst release, a low reaction temperature, a good yield of product, and the simplicity of the procedure. Furthermore, milder reaction conditions, simple set-up, shorter reaction time, great deal with clean protocols, and the absence of solvents are all advantages of this methodology, making it an appealing alternative to existing methods. The catalyst easily separated by simple filtration and reused at least five times with just a minor drop in catalytic activity [31].

In earlier 2019, Kerru et al. reported a highly efficient facile green method is one-pot multicomponents condensation of 1,2,4,5-tetraryl imidazoles compounds by employing environmental benign easily available inexpensive red brick clay powder as heterogeneous catalyst [Table 3, Entry 3] under in aqueous medium at 60 °C. In this methodology noticeable advantage of water as medium opens a new attractive approach for green and rapid synthesis of desired compounds with good yields. No column chromatography and ease of product purification are the other benefits of this protocol. Therefore, the red brick powder is a cost-effective and potential green catalyst in organic synthesis. This can help to develop catalytic processes while lowering environmental effect [32].

In 2018, Magyar et al. discovered that Ti^+4^ on a 4A° molecular sieve support as an excellent catalyst [Ti^+4^/4 A° MS, Table 3, Entry 4] for the one-pot, three-component synthesis of 2,4,5-triaryl imidazoles at 100 °C under mild basic conditions. The efficient heterogeneous catalyst recovered readily and was reused in three additional runs without a significant drop in activity using the proposed technique. After the simple workup, the desired product obtained in 80% yield. Notable disadvantage of this methodology used toluene is harmful for health and product obtained in average in yield [7].

In 2017, Selvakumar et al. used a heteropoly-11-tungsto-1-vanadophosphoric acid (HPV) supported on activated natural clay catalyst [HPVAC-20, Table 3, Entry 5] for the one-pot, three component synthesis of 2,4,5-triaryl and 1,2,4,5-tetraryl imidazoles under solvent-free reaction conditions at 120 °C. Green heterogeneous reaction conditions, a simple workup approach, a fast reaction time, good product yields, and an effective and reusable catalyst for four successive cycles make this approach more commercially feasible and environmentally benign [33].

Fatemeh ‘s group achieved a simple, highly versatile, and efficient synthesis of 2,4,5-triaryl and 1,2,4,5-tetraryl imidazole compounds in 2014 using a new eco-friendly solid acid catalyst made by reacting kaolin with chlorosulfonic acid [Kaolin-SO_3_H, Table 3, Entry 6] at a moderate temperature under solvent-free environment. Shorter reaction durations, good yields, easy handling and quick work-up, and easy availability of cheaper natural based catalyst is all notable features in this study. This approach does not investigate the catalyst’s reusability [34].

In 2013, Xu et al. attempted to develop a convenient method for the synthesizing of the 2,4,5-triaryl imidazoles to use a catalytic quantity of ferric(III) nitrate endorsed on kieselguhr [Fe(NO_3_)_3_-Kie, Table 3, Entry 7] as an effective and relatively inexpensive Lewis acid catalyst in a solvent-free environment at 120 °C. Environmentally friendly approach, shorter response periods, easy work-up process, use of nontoxic catalyst, and facile isolation and recycling of the catalyst are just a few of the benefits of this methodology. The solid catalyst was easily isolated from the reaction mixture, and Ferric (III) nitrate, kieselguhr commercially available and employed without additional purification; the process appears to be very cost-effective [35].

In 2013, Kannan et al. performed K10 supported titanium catalyst [K10Ti, Table 3, Entry 8]. The catalyst was found to be an effective for the synthesis of 1,2,4,5-tetraryl imidazole derivatives under the neat condition at 120 °C. This technology not only produces good yields, but it also eliminates issues such as handling, safety, and pollutants. This catalyst is nonvolatile, recyclable, nonexplosive, and easy to handle, making it ideal for a range of organic transformations. The proposed catalyst is thermally stable and the catalyst recyclability is found to be steady across three cycles with no discernible activity reduction [36].

Sivakumar et al. reported another green chemistry related protocol in 2010, have achieved novel, mild, and efficient method for the multicomponent condensation reactions, one-pot eco-friendly synthesis of 2,4,5-triaryl and 1,2,4,5-tetraryl imidazoles employing zeolite supported metal nitrates [Cu (NO_3_)_2_-Zeolite, Table 3, Entry 9] in absence of the solvent at 80 °C. This synthesis process is interesting because it is associated with one or more advantages such as ease of operation, affordable catalysts, good yield of products, and the use of nontoxic catalyst. Cu(NO_3_)_2_ is a supported metal nitrate that has gotten a lot of attention due of its acceptable acidity, nontoxicity, ease of availability, and low cost, making it a viable table top reagent [37].

In 2009, Gadekar et al. utilized inexpensive natural scolecite [Table 3, Entry 10] as an effective catalyst for the one-pot synthesis of 2,4,5-triarylimidazole derivatives via a three component reaction using benzil or benzoin, aldehydes and ammonium acetate in ethanol. Scolecite is an affordable, nontoxic, and recyclable natural zeolite with Lewis and Bronsted acidic sites that allow it to operate as a bifunctional heterogeneous catalyst in an environment-friendly manner. This methodology has a number of advantages, including mild reaction conditions, reusability, quick reaction speed, cleaner reactions, high product yields, and simple experimental and isolation procedures, all of which make it a useful and appealing method for the synthesis of 2,4,5-triarylimidazole derivatives [38].

**Scheme 4:**
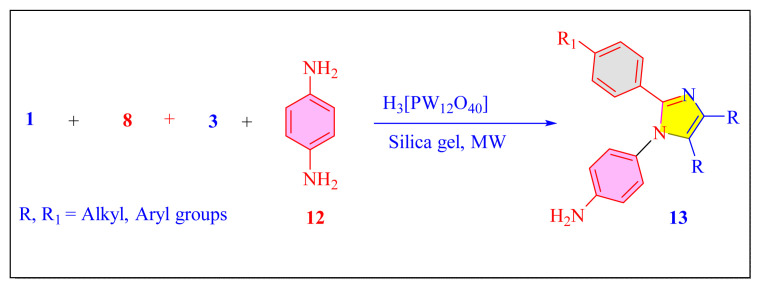
Synthesis of 1,2,4,5-tetrasubtituted imidazoles derivatives.

In 2016, Yan et al. have evaluated one-pot multicomponent condensation of 4-amino, aryl diketone, benzaldehyde derivatives of auxochrome and ammonium acetate in the presence of solid support SiO_2_ gel and Keggin-H_3_[PW_12_O_40_] catalyst [Table 4, Entry 1] under the microwave irradiation and solvent-free condition [Scheme 4]. This method focused on luminescence properties of the synthesized tetrasubstituted imidazole compounds. There are some limitations to this process in terms of reagent cost, average product yield, and catalyst recyclability [39].

Das et al. in 2013 have investigated the comparison of the two catalyst performances such as secondary amine based ionic liquid [[IL= [n-Pr_2_NH_2_] [HSO_4_] and defective Keggin class heteropolyacid [HPA = H_6_PAlMo_11_O_40_, Table 4, Entry 2] are separated use for effective one-pot multicomponent synthesis of 1,2,4,5-tetrasubstituted imidazoles. This approach also displays the use of urea as a nitrogen supply instead of the commonly used ammonium acetate, which results lower cost. Excellent yield, operational flexibility, quick response time, solvent-free circumstances, including the use of nontoxic, and environmentally benign accelerators are just a several of the innovative key features. The comparative results in present method product yield of the reactions with HPA are higher over IL reactions [40].

In 2012, Kalkhorani et al. developed an effective and simple approach for one-pot synthesis of multisubstituted imidazole derivatives in solvent-free conditions utilizing K_7_Na_3_P_2_W_18_Cu_4_O_68_ as a catalyst at 140 °C [Table 4, Entry 3]. Noticeable advantages of this method are easy to handle and useful catalyst, better productivity, simple work-up, and nonchromatographic compound purification (crystallization only). This approach is also harmless to the environment. For heterogeneous catalysts, reusability is an essential aspect. The catalyst was separated by simple filtration and cleaned with CH_2_Cl_2_ after the first run of the reaction, then kept to dry at 100 °C for 1 h before being utilized for comparable reactions in 5 runs with no significant loss in activity [41].

Rafiee et al. reported in 2011 that a 40% phosphotungstic acid supported on silica showed greater efficacy [40% PWA/SiO_2_, Table 4, Entry 4] in the synthesis of tetrasubstituted imidazoles under solvent-free conditions at 140 °C. The yields were excellent, with no side products such as trisubstituted imidazoles, which are generally formed when strong acids are present. Simple experimentation, recyclable catalyst, excellent product yields, minimal reaction time, and no toxic solvent are all advantages. Furthermore, the suggested catalyst is heat resistant, environmentally friendly, uncomplicated, and affordable to make, and it can be easily isolated from reaction mixtures and reuse multiple times, all of which are strong arguments for considering its application in industrial and commercial applications. The preparation of catalysts is subject to some constraints, such as stirring the final catalysts mixture overnight at room temperature [42].

In 2009, Karimi et al. identified a simple and efficient method for synthesizing 2,4,5-trisubstituted and 1,2,4,5-tetrasubstituted imidazole compounds using Wells–Dawson (WS) heteropolyacid (H_6_P_2_W_18_O_62_•24H_2_O) supported on silica [WD/SiO_2_, Table 4, Entry 5] as an effective catalyst under solvent-free environments at microwave-assisted (MW) or conventional heating. The current methodology has a number of appealing aspects, including a simple operation, shorter reaction times, better productivity, convenience of recovery and recycling of the catalyst, and commercial feasibility of the catalyst, all of which combine to make this method a cost-effective, environmentally friendly, and waste-free chemical compound [43].

**Scheme 5:**
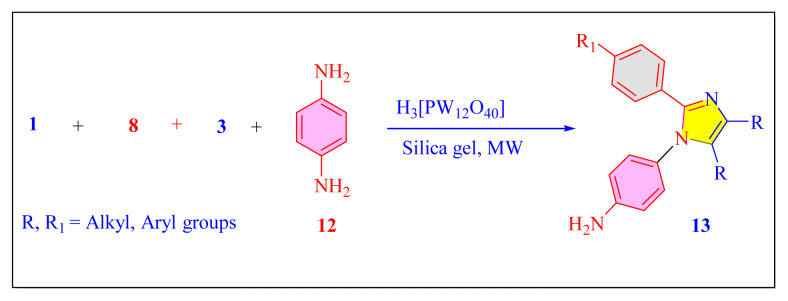
Synthesis of 2,4,5-triaryl imidazole derivatives.

In 2008, Heravi et al. commented on the synthesis of 2,4,5-triaryl-imidazoles from the condensation with benzaldehyde, NH_4_OAc, and 1,2-diketone in the presence of HPAs as efficient catalysts (H_14_[NaP_5_W_30_O_110_], Table 4, Entry 6) in acetonitrile at reflux conditions [Scheme 5]. The said protocol shoed the benefits, which include good yields with excellent purity, a quick reaction time, catalysts retrieved by filtration and reused after recovery [44].

Heravi et al. in 2006 have explored Keggin class heteropolyacid [H_4_PMo_11_VO_40_, Table 4, Entry 7] under reflux in ethanol, is an extremely effective solid acid catalyst for synthesis of 1,2,4,5-tetrasubstituted imidazoles compounds. Greater yields, relatively fast reaction times, ease of operation and work-up, and the desired products separated by simple filtration are some of the benefits of this approach. This approach works with both aliphatic and aromatic amines to make tetrasubstituted imidazoles. HPAs (heteropolyacids) have a number of benefits that make them economically and environmentally appealing in both research and commercial contexts [45].

In 2011, the group of Naser have performed a very simple, mild and efficient methodology for the preparation of 1,2,4,5-tetrasubstituted imidazoles through one-pot multicomponent reaction by using SiO_2_ gel support on poly phosphoric acid [PPA-SiO_2_, Table 4, Entry 8] in the absence a solvent and microwave irradiation conditions. This method outperforms other methods in terms of yield, reaction time, ease of set-up, and the absence of volatile and toxic organic solvents. Additional benefits of this approach include ease of use and environmental benefits due to the lack of harmful catalysts and solvents. The catalyst can be reused at least four times with just a minor drop in catalytic activity [46].

In 2016, Thimmaraju et al. achieved efficient synthesis of 2,4,5-trisubstituted imidazole derivatives within solvent-free condition at intermediate temperature employing green ZrO_2_-Al_2_O_3_ as an effective catalyst [Table 4, Entry 9]. The surface acidity of solid supported ZrO_2_-Al_2_O_3_ catalysts prepared by impregnation method is higher than that of ZrO_2_-Al_2_O_3_ catalysts prepared by solution combustion or precipitation method. The current approach is straightforward in terms of experimentation, has a quicker reaction time, is nontoxic, and uses economical reagents, cleaner reaction routes, and an environmentally friendly catalyst. The catalytic components easily separated from the reaction mixture. They can be recycled up to six times without losing their potency Catalytic material was filtered from the reaction mixture, cleaned by acetone, dry at 120 °C for 1 h, and calcined at 550 °C for 0.5 h, which is a noteworthy observation of this approach [47].

Green chemistry approach for the synthesis of 2,4,5-substituted as well as 1,2,4,5-substituted imidazoles using acidic Al_2_O_3_ penetrated with ammonium acetate [Al_2_O_3_-NH_4_OAc, Table 4, Entry 10] as the solid support with solvent-free and microwave-assisted synthesis was reported by Ya et al. in 2000. High yields, fast reaction times, ease of operation and an environmentally friendly catalyst are all advantages of this technique. In the present work further expand the scope of reactions applicable to MICROCOS (microwave-assisted combinatorial synthesis) technology by introducing the microwave-assisted synthesis of diverse substituted imidazoles [48].

## 3. Polymer supported nanocatalysts in imidazole synthesis

Mohtasham et al. in 2021 have reported new magnetically separable horsetail plant ash as a natural resource of mesoporous silica-supported to the magnetite to make of Fe_3_O_4_@HA as an extremely powerful solid acid nanocatalyst [Fe_3_O_4_@HA, Table 5, Entry 1] for the synthesis of trisubstituted imidazole derivatives at room temperature in aqueous medium. The key features of this method are including green catalyst with natural source, outstanding activity and stability, simple and efficient isolation using an external magnet, resulting in minimal catalyst loss during separation. Furthermore, the catalyst is recovered and recycled six times with no discernible loss of reactivity or yields, makes current synthesis more efficient and ecologically sustainable [49].

In 2021, Varzi et al. have introduced green and sustainable approach for one-pot multicomponent synthesis of substituted imidazole derivatives by employing novel nature based efficient hybrid nanocatalyst comprised by “guarana” as a natural polymeric basis supported into magnetite and copper(I) oxide nanoparticles [Cu_2_O/Fe_3_O_4_@guarana, Table 5, Entry 2] with ultrasound wave irradiation at room temperature. Polymeric textures made from natural materials are often favoured in this technique because of their biocompatibility, biodegradability, and nontoxicity, all of which match the green chemistry principles. Furthermore, this approach well illustrated by high reaction yields obtained in the presence of a modest amount of these nanocomposites under mild circumstances, a large active surface area, significant magnetic behaviour, great heterogeneity, acceptable stability, and reusability. Additionally, this nanocatalyst has a remarkable recyclability, allowing it to be reused six times without a significant drop in catalytic efficiency [50].

**Figure 6:**
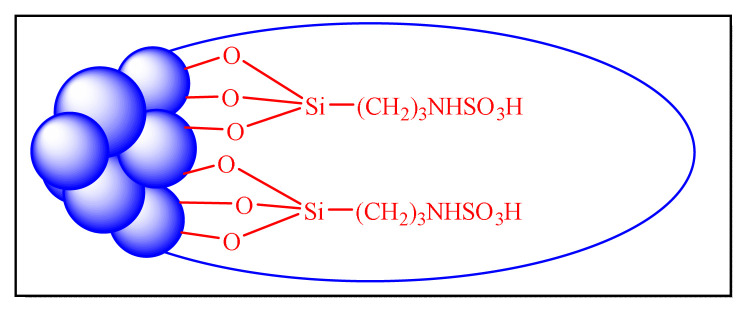
HMS-SA solid acid catalyst.

In 2020, Arora et al. have demonstrated green and efficient sulfamic acid functionalized hollow magnetically retrievable solid-acid catalyst [Figure 6, HMS-SA, Table 5, Entry 3] employed for the synthesis of trisubstituted imidazole compounds through one-pot multicomponents condensation reaction with ultrasonic irradiation. Eco-friendly synthesis, mild reaction conditions, outstanding yields, good selectivity, quick reaction times, use of ultrasound as a green energy, less wastage, and easy magnetic recovery and recycling of the catalyst are some of the distinguishing features of the current protocol over existing routes. The catalyst was easily recover from the reaction medium and displayed good recyclability for nine runs with no noticeable reduction in catalytic activity. In this approach appears to be less impressive due to the usage of hazardous toluene solvent in the solid acid catalyst preparation [51].

Varzi et al. presented a new bimetallic Lewis acid, ZnSZnFe_2_O_4_ [Table 5, Entry 4], as an excellent heterogeneous magnetic nanocatalyst for the synthesis of 2, 4,5triaryl-1H-imidazole derivatives with ultrasonic irradiation in ethanol at conventional heating in 2019. The benefits of this technique include milder reaction condition, excellent product yields, and a simple workup method. ZnS-ZnFe_2_O_4_ MTMO applied as an efficient nanocatalyst in multicomponent and showed superior activity in terms of time, temperature and even the efficiency in comparison with other reported catalysts such as montmorillonite K10, Yb (OPf)_3_ and Zr (acac)_4_. Catalyst recyclability is six times for future reactions without a substantial loss of activity, and it can be recovered using only externally magnets [52].

**Figure 7:**
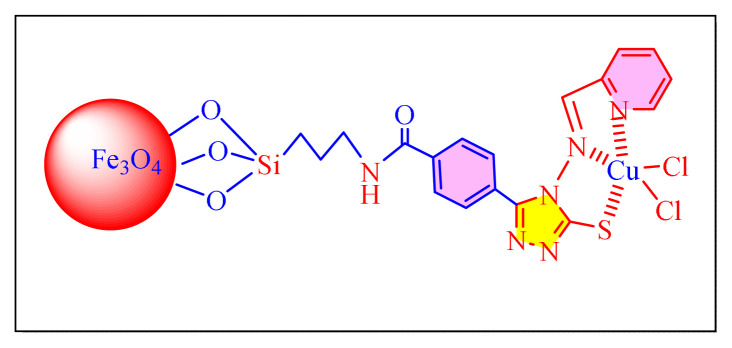
Cu@imine/Fe_3_O_4_ MNPs catalyst.

Using Cu@imine/Fe_3_O_4_ magnetic nanoparticles (MNPs) as a catalyst, Thwin et al. provided a comprehensive, simple, and successful procedure for multicomponent tetrasubstituted imidazole compounds [Figure 7, Table 5, Entry 5] under solvent-free conditions at classical heating in 2019. Low catalytic loading, quick reaction durations, facile separation and purification of the compounds, and excellent yield are all advantages of this approach. Furthermore, the catalyst recovered and recycled six times with no discernible loss of reactivity or yields, making the new synthetic process more practical and environmentally beneficial. Although, the methodology seems to be successful, the preparation of catalyst involved many steps which the credit of the methodology [53].

Kalhor et al. in 2019 have proposed a novel magnetic solid acid catalyst like SO_3_H@zeolite-Y nanocomposites supported nano-Fe_3_O_4_ [Fe_3_O_4_/SO_3_H@zeolite-Y, Table 5, Entry 6] for the synthesis of 2,4,5-triarylsubstituted imidazole derivatives in ethanol as solvent by conventional heating. The most interesting features from the perspective of environmental remediation, the current technique has several advantages, including high yield, purity of products, nontoxic nature of the nanocatalyst, reusability of the nanocatalyst, fast reaction rate as well as economical chemical process. An external magnetic force can easily recycle the nanocatalyst, which can be reused up to five times without losing its potency [54].

Afruzi et al. in 2019 have utilized cost-effective hybrid heterogeneous catalyst ZnS/CuFe_2_O_4_ [Table 5, Entry 7] in the synthesis of 2,4,5-triaryl imidazole derivatives in ethanol with classical heating. This approach has various advantages, such as good yield, mild reaction conditions, and a straightforward set-up. Catalyst that seems to be cost-effective and it can be used five times in subsequent reactions without losing catalytic performance [55].

In 2019, Gilan et al. achieved an effective nanomagnetic catalyst on supported Fe_3_O_4_ core with the N-Ligand Co complex [(Fe_3_O_4_@SiO_2_@Si-(CH_2_)_3_@N-Ligand@Co MNPs, Table 5, Entry 8] for the synthesis of multicomponent tetrasubstituted imidazole derivatives from the one-pot condensation reaction under solvent-free condition. In this protocol, nanomagnetic heterogeneous catalyst can reduce the time and increase the yield of reaction at optimum reaction condition confirmed by design of experiment method and the response surface methodology used to the optimization of the reaction conditions. The technique introduced new magnetic nanocatalyst with efficient catalytic performance though it involved longer and multistep preparation of catalyst [56].

Maleki et al. in 2019 have performed a new approach for preparation of green and inexpensive Bronsted acid heterogeneous nanocatalyst by preparation of Fe_3_O_4_@PVA nanostructure after its sulfonation [Fe_3_O_4_@PVA-SO_3_H, Table 5, Entry 9] in the synthesis of 2,4,5-trisubstituted-1H-imidazole compounds refluxing in ethanol. Short reaction times, great yields, and recyclability of the catalyst for ten consecutive rounds were all highlighted in the approach, with no appreciable loss in catalytic performance [57].

**Figure 8:**
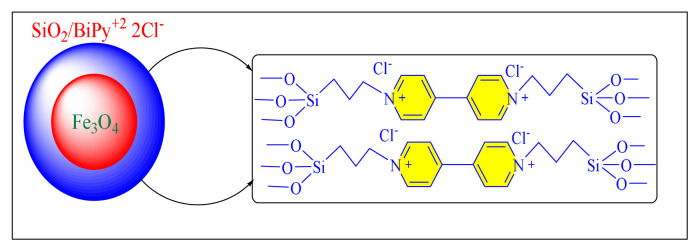
Fe_3_O_4_@SiO2/BiPy^+2^ 2Cl^−^ heterogeneous nanocatalyst.

Hosseini et al. in 2019 have reported green and an efficient magnetically separated nanocatalyst comprised of bipyridinium support on silica-coated magnetite nanoparticles were successfully produced [Fe_3_O_4_@SiO_2_/BiPy^+2^ 2Cl^−^, Figure 8, Table 5, Entry 10]. The catalytic activity was investigated using one-pot cyclocondensation procedure to synthesise several tri/tetrasubstituted imidazole compounds in solvent-free condition at 120 °C. Fe_3_O_4_@SiO_2_/bipyridinium nanocatalyst has a superior efficiency than the other comparable catalysts. The technique provides multiple benefits, including good yield, ease of operation, shorter reaction duration, clean processing conditions, and the catalyst’s renewability. As a result, the Fe_3_O_4_@SiO_2_/bipyridinium catalyst was recovering from a reaction mixture and employed in five cycles with almost negligible activity loss [58].

In 2018, Gilan et al. used egg shell as a waste material packed on a nano-Fe_3_O_4_ surface ( Fe_3_O_4_@eggshell) to transform it to Fe_3_O_4_@Ca_3_(PO_4_)_2_ [Table 5, Entry 11] as a nanomagnetic catalysts for the one-pot multicomponent synthesis of tetrasubstituted imidazole derivatives in a solvent-free environment. The catalyst systems were reported to be reusable during four consecutive cycles, with a gradual decrease in activity after each cycle. Despite the fact that it required a multistep catalyst preparation [59].

**Figure 9:**
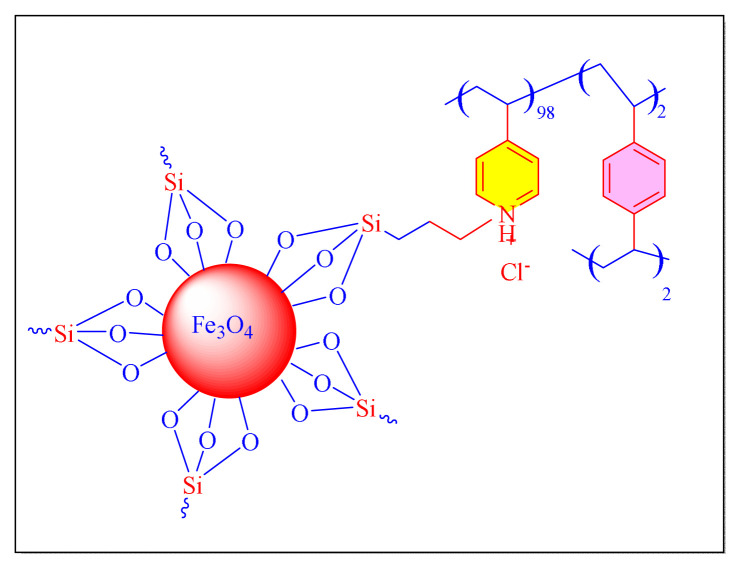
[P_4_-VP]-Fe_3_O_4_ MNPs catalyst.

Hamidi et al. in 2018 have validated crosslinked poly(4-vinylpyridine) supported Fe_3_O_4_ nanomagnetic particles for the synthesis of substituted imidazole compounds in the absence of solvent condition [[P_4_-VP]-Fe_3_O_4_NPs, Figure 9, Table 5, Entry 12]. The catalytic systems appear to be reusable numerous times without appreciable reduction in quality. The catalyst preparation took longer time, restricting the methodology’s creditability [60].

In 2017, Korani et al. applied a novel green and efficient tri-iodide modified magnetic nanocatalyst [Fe_3_O_4_@SiO_2_@(CH_2_)_3_N^+^Me_3_I_3_^−^, Table 5, Entry 13] for the synthesis of imidazole compounds in absence solvent at 120 °C. After each rerun, the magnetically recovered nanocatalyst reused during five more cycles, with the catalytic activity gradually decreasing [61].

**Scheme 6:**
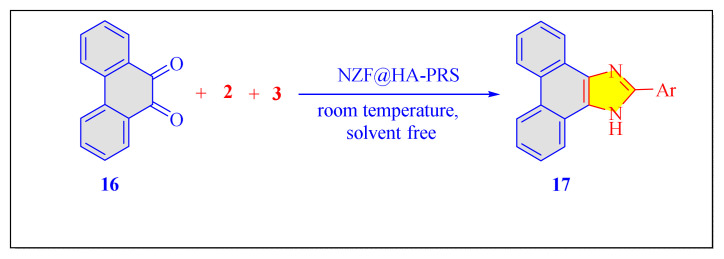
Synthesis of imidazole compound in presence of NZF@HA-PRS nanocatalyst.

The deployment of Preyssler heteropolyacid (HPA) bound on magnetic nanoparticles as a sustainable and recyclable catalysts for synthesis of imidazole compounds was reported from Javid’s group in 2017 [NZF@HA-PRS, Table 5, Entry 14] under solvent-free conditions at ambient temperature, using a one-pot three-component condensation process of aromatic aldehydes, [9,10]-phenanthraquinone, and ammonium acetate [Scheme 6]. Efficacy of [NZF@HA-PRS] nanocatalyst at the room temperature found remarkable part of the protocol [62].

Ahooie et al. in 2017 have accomplished environmental friendliness methodology to the synthesis of trisubstituted imidazoles with one-pot multicomponent condensation reaction in presence of reusable magnetically graphitic carbon nitride [Fe_3_O_4_@g-C_3_N_4_, Table 5, Entry 15] nanocomposite as catalyst by using ethanol as solvent. The approach is remarkable for the benefits in the synthesis of different imidazole derivatives due to the use of green solvent, moderate to excellent yields. Significantly, the nontoxic and affordable Fe_3_O_4_@g-C_3_N_4_ magnetic nanocatalyst exhibited remarkable recycling process employing an external magnet, with no decrease in parental catalytic efficacy after 10 cycles [63].

In 2017, Zolfagharinia et al. demonstrated novel magnetic nanocatalyst of phosphomolybdic acid (PMA) immobilization of on the surface of core-shell zirconia-coated with ferrous ferric oxide (FeO_3_O_4_) magnetic nanoparticle [Fe_3_O_4_@ZrO_2_/PMA, Table 5, Entry 16] for the one-pot multicomponent condensation reaction for the synthesis of 2,4,5- trisubstituted and 1,2,4,5-tetrasubstituted imidazoles derivatives. The catalyst activity was evaluated in refluxing solvent as well as solvent-free conditions, solvent-free conditions showing better yields in quicker reaction times [64].

**Figure 10:**
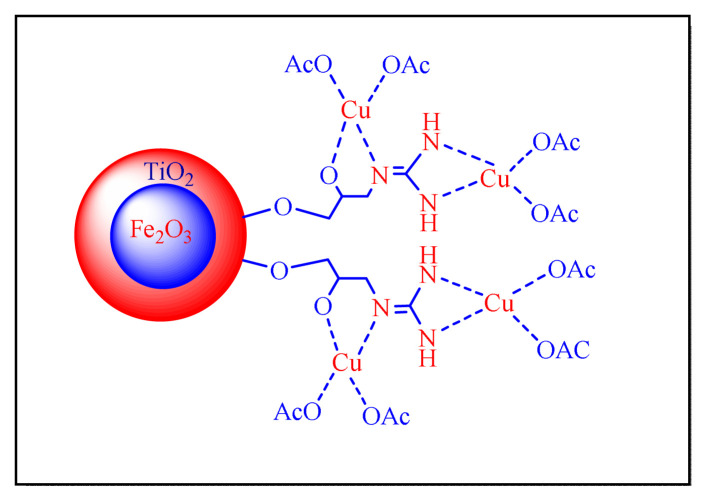
γ-Fe_2_O_3_@TiO_2_-EG-Cu (II) MNPs nanocatalyst.

Nejatianfar et al. published a new and effective method for synthesising 2,4,5 trisubstituted and 1,2,4,5 tetrasubstituted imidazoles in 2017 by using Cu(II) supported to guanidinated epibromohydrin-functionalized to titanium oxide shell with core of ferrous ferric oxide (Fe_2_O_3_) magnetic nanoparticle as catalyst [γ-Fe_2_O_3_@TiO_2_-EG-Cu(II), Figure 10, Table 5, Entry 17] in solvent-free circumstances. Formation of the catalyst is a magnetite core and a titanium oxide shell; the mean dimensions are 10–20 nm and 5–10 nm, respectively. The nanocatalyst can be reused during at least six times without decreasing its catalytic efficiency. This protocol seems conventional and less impressive due use the of toxic dry toluene solvent during the preparation of nanocatalyst [65].

**Scheme 7:**
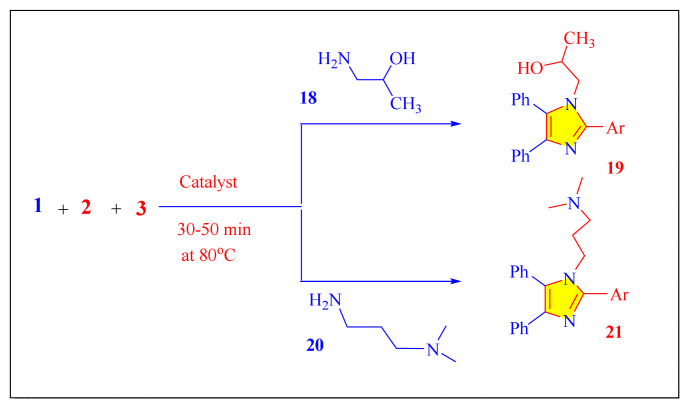
Synthesis of multisubstituted imidazoles derivatives.

Marzouk et al. in 2017, have applied environmental friendly novel ZnFe_2_O_4_ magnetic nanocatalyst with one-pot multicomponent condensation synthesis of multisubstituted imidazoles by using the benzil with various aromatic aldehydes, ammonium acetate and aliphatic amines [1-amino2-propanol (18) and N,N’-dimethyl-1,3-propanediamine (20)] under neat conditions [Scheme 7, Table 5, Entry 18]. Furthermore, ZnFe_2_O_4_ magnetic nanoparticles have thermal and chemical stability and can be used in at most of five consecutive cycles no significant activity loss [66].

**Figure 11:**
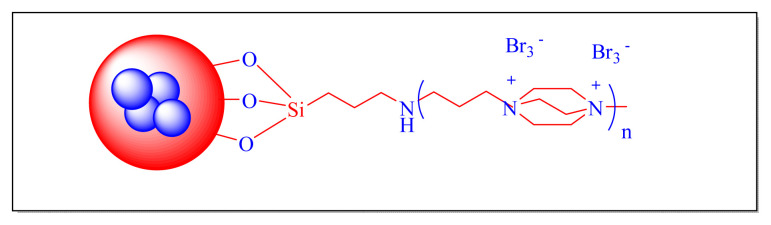
Fe_3_O_4_@SiO_2_@polyionene/Br_3_^−^ magnetic core-shell nanocatalyst.

**Scheme 8:**
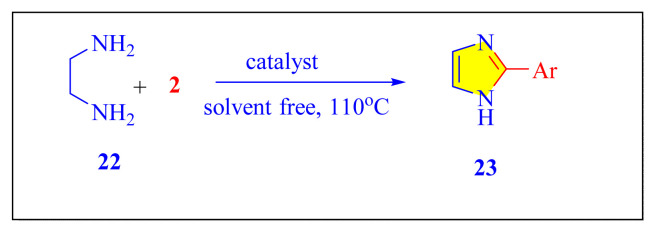
Synthesis of imidazoles derivatives by using Fe_3_O_4_@SiO_2_@polyionene/Br_3_^−^ magnetic core-shell nanocatalyst.

In 2016, Dezfoolinezhad et al. demonstrated new eco-friendly method to synthesize imidazole compounds using an aldehyde and 1,2-diaminoethane in presence of magnetic core shell Fe_3_O_4_@SiO_2_@polyionene/Br_3_^−^NPs [Scheme 8, Figure 11, Table 5, Entry 19] with classical heated at 110 °C under neat condition. Many steps are involved in the preparation of the catalyst in this procedure, and the time required is more than six days, which seem to be limitations of this methodology [67].

In 2016, Sanasi et al. attempted the synthesis of 2,4,5-trisubstituted and 1,2,4,5-tetrasubstituted imidazole compounds employing spinel copper ferrite as a nanocatalyst [CuFe_2_O_4_, Table 5, Entry 20] under ultrasonic irradiation at room temperature in ethanolic medium. The catalysts are magnetically separable and recyclable. They have reused five cycles in a row with no significant loss of catalytic activity. Despite the fact that the proposed process involved sonication at ambient temperature, it was successful in reducing reaction time while also increasing yields to excessive levels [68].

**Figure 12:**
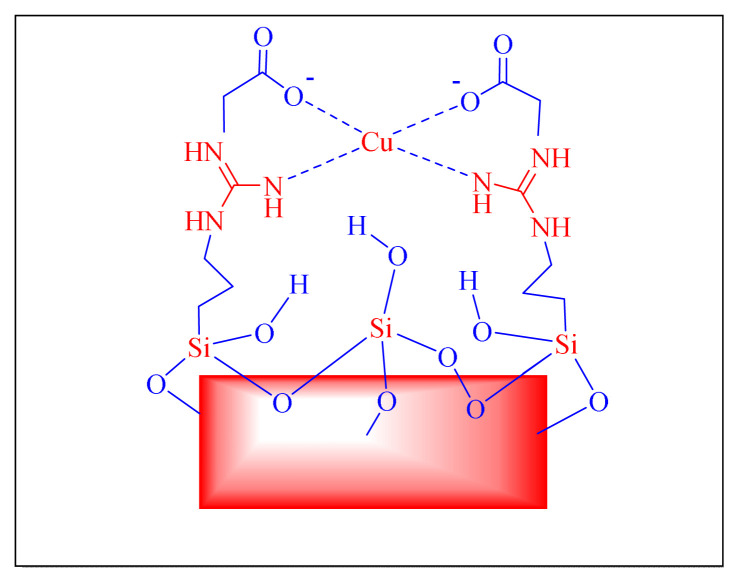
Cu/GA/Fe_3_O_4_@SiO_2_ magnetic nanocatalyst.

In 2016, Shaabani et al. proposed an efficient and eco-friendly nanocatalyst for the one-pot multicomponent synthesis of trisubstituted imidazole derivatives under neat conditions, nanocatalyst was prepared through the ultrasonic-assisted linking of guanidine acetic acids on adapted Fe_3_O_4_@SiO_2_ core-shell nanomaterial’s spheres and successive immobilized to Cu (II) [Figure12, Cu/GA/Fe_3_O_4_@SiO_2_, Table 5, Entry 21]. The approach featured the introduction of a new silica-supported catalyst that produced outstanding product yields and had quick reaction times. This methodology protocol is remarkable for so many of the steps involved in the preparation of catalyst, as well as the time required, which is more than three days, which are limitations [69].

In 2015, Maleki et al. presented effective and environmental friendly nanocatalyst, which are prepared by the phosphomolybdic acid (H_3_PMo_12_O_40_) supported into the silica and magnetic coated NiFe_2_O_4_ nanoparticals [NiFe_2_O_4_@SiO_2_–H_3_PMo_12_O_40_, Table 5, Entry 22] for the preparation of trisubstituted as well as tetrasubstituted imidazole compounds. Furthermore, a magnet strip readily separated the catalysts from the reaction medium, allowing it to be reused at most ten times no substantial loss of activity [70].

Zarnegar et al. in 2014 have demonstrated highly effective and novel heterogeneous nanocatalyst Fe_3_O_4_–polyethylene glycol–Cu nanocatalyst [Fe_3_O_4_–PEG–Cu, Table 5, Entry 23] to the synthesis of tri/tetrasubstituted imidazole derivatives under neat condition by classical heating at 110 °C. The authors reported high yields of corresponding imidazoles with excellent reusability of magnetically recovered catalyst [71].

**Figure 13:**
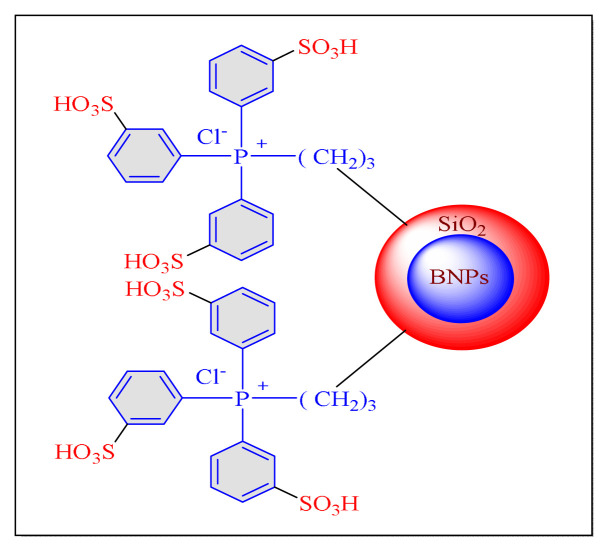
BNPs@SiO_2_-TPPTSA nanocatalyst.

In 2020, Khodamorady et al. demonstrated a low-cost raw materials approach for boehmite nanoparticles (BNPs) as a solid substrate coated with silica and anchored over triphenylphosphine trisulphonic acid (TPPTSA) for the homoselective synthesis of trisubstituted imidazole compounds under neat condition using conventional heating [Figure 13, BNPs@SiO_2_-TPPTSA, Table 6, Entry 1]. Due to the lengthier catalyst construction time (more than two days) and the usage of hazardous chemicals (dry toluene), the current protocol is less noteworthy [72].

**Figure 14:**
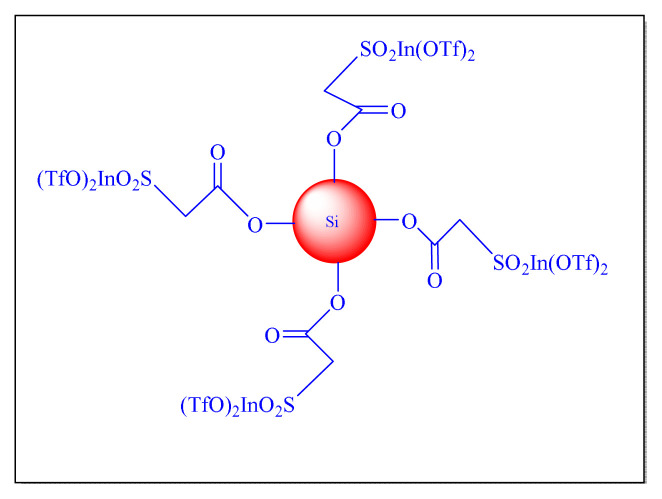
SiSAIn(OTf)_2_ nanocatalyst.

In 2019, Vaid et al. have proposed sulfoacetate modify SiO_2_ immobilized indium (III) triflate [SiSAIn(OTf)_2_, Table 6, Figure 14 Entry 2] as an efficient solid acid heterogeneous nanocatalyst. The reusability of the catalyst SiSAIn (OTf)_2_ was reported for several cycles with no significant activity reduction. In highlight of the present protocol seems conventional and less impressive due to the use of toxic solvent during preparation of nanocatlyst [73].

Navamani et al. in 2017 have performed novel multicomponent condensation of the 2,3-diketobutane, ammonium acetate, substituted aromatic aldehydes and substituted aniline for the synthesis of the tetrasubstituted imidazole derivatives in ethanol medium by using nano-SiO_2_ [Table 6, Entry 3] as a highly efficient catalyst at 80 °C [Scheme 09]. The procedure has successfully added new kind of substituted imidazoles, but it provides no information on nanocatalyst reusability [74].

In 2016, Esmaeilpour et al. evaluated role of Dendrimer-PWAn nanoparticals [Table 6, Entry 4] as efficient, green and environmental friendly catalyst for the synthesis of 2, 4, 5-trisubstituted as well as 1, 2,4,5-tetrasubstituted imidazole derivatives with one-pot condensation reaction. When compared to conventional heating in solvent-free circumstances to their current technology using ultrasonic irradiation provided equivalent yields of products with shorter reaction durations. The catalyst can be removed easily from the products using filtration, as well as the retrieved catalysts can be recycled for up to six cycles without losing activity [75].

**Figure 15:**
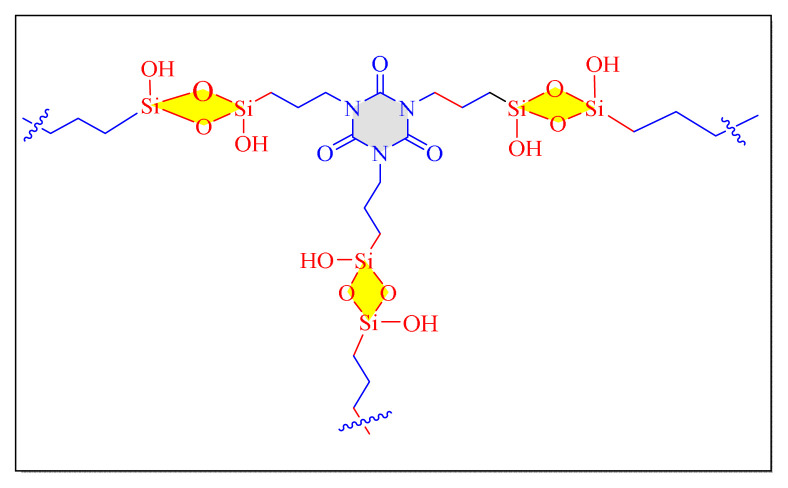
PMO-ICS nanocatalyst.

Dekamin et al. in 2016 have attempted one-pot multicomponent synthesizing of 2,4,5-trisubstituted imidazoles compounds by using PMO-ICS nanocatalyst (Isocyanurate bridging periodic mesoporous organosilica) reflux in ethanol [PMO-ICS, Figure 15, Table 6, Entry 5]. As a result, minimal catalyst loading, avoidance of hazardous transition metals or sensitive reagents for catalytic activity modification, are all appealing aspects of this approach. The reusability of catalyst for four more consecutive cycles were performed and showed almost retained catalytic activity. The present protocol seems conventional and less impressive due preparation of catalyst time required is more than five days [76].

In 2015, Nikoofar et al. investigated simple and efficient concentrated nitric acid with nano-SiO_2_ as a support [HNO_3_-nano-SiO_2_, Table 6, Entry 6] catalyst for the synthesis of several tri- and tetrasubstituted imidazole derivatives under neat conditions at 100 °C. With each recycles, the reusability of the retrieved catalyst mentioned for three more successive cycles with no notable reduction in performance [77].

**Figure 16:**
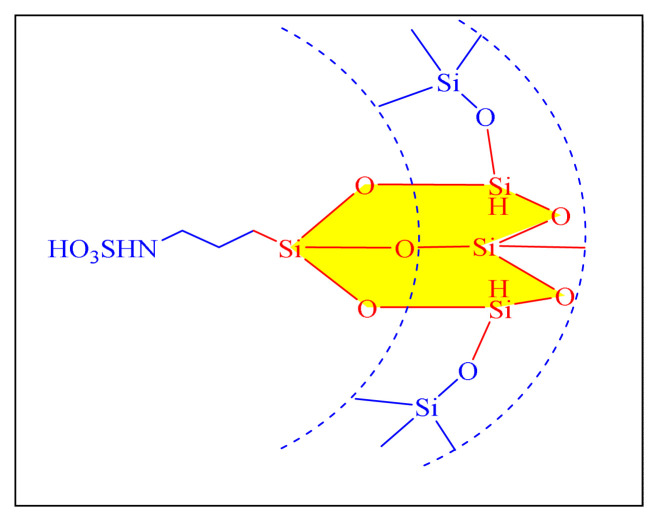
NHSO_3_H-KIT-5 nanocatalyst.

Mirsafaei et al. in 2015 have proposed an eco-friendly one-pot multicomponent synthesis of 2,4,5-trisubstituted imidazoles by employing silica-bonded propyl-N-sulfamic acid heterogeneous nanocatalyst [NHSO_3_H-KIT-5, Figure 16, Table 6, Entry 7]. KIT-5, a silica class mesoporous material with the relatively high surface areas (1090 m^2^ g^−1^), has been used to make a simple and effective catalyst. This approach contributed a new form of nanocatalyst for the improvement in the imidazoles synthesis. The use of toxic solvent like toluene during the preparation of catalyst limits the credit of methodology [78].

In 2015, Bakavolia et al. described an effective and environmental friendly method for the synthesis of 2,4,5-trisubstituted imidazole derivatives by using SiO_2_ support ferric hydrogen sulphate (FHS) nanocatalyst [SiO_2_-FHS, Table 6, Entry 8] in the solvent-free condition at 110 °C. The catalyst’s recyclability was indicated for four successive cycles, with considerable loss in catalytic performance observed as a considerable limitation part of the protocol [79].

In 2014, Sharghi et al. have evaluated a novel Mo (IV) salen complex supported with 3-aminopropyl functionalized silica gel nanomaterials as heterogeneous nanocatalyst [Table 6, Entry 9] to the synthesized 2,4,5-trisubstituted imidazole derivatives in the ethanol as solvent at 50 °C. The attributes of this method make our procedure convenient, mild reaction condition, low cost, green, and efficient. The recovered catalysts successfully recycled five times in a row with no noticeable reduction in quality [80].

Gharib et al. in 2013 have reported an efficient method in order to synthesize a variety of 2,4,5-trisubstituted as well as 1,2,4,5-tetrasubstitued imidazole derivatives in the absence of solvents at reflux condition by using silica-supported heteropolyacid (Preyssler) as a nanocatalyst [H_14_[NaP_5_W_30_O_110_]-SiO_2_, Table 6, Entry 10]. The method claimed high economy of yields, reduced reaction times and catalyst reusability [81].

**Figure 17:**
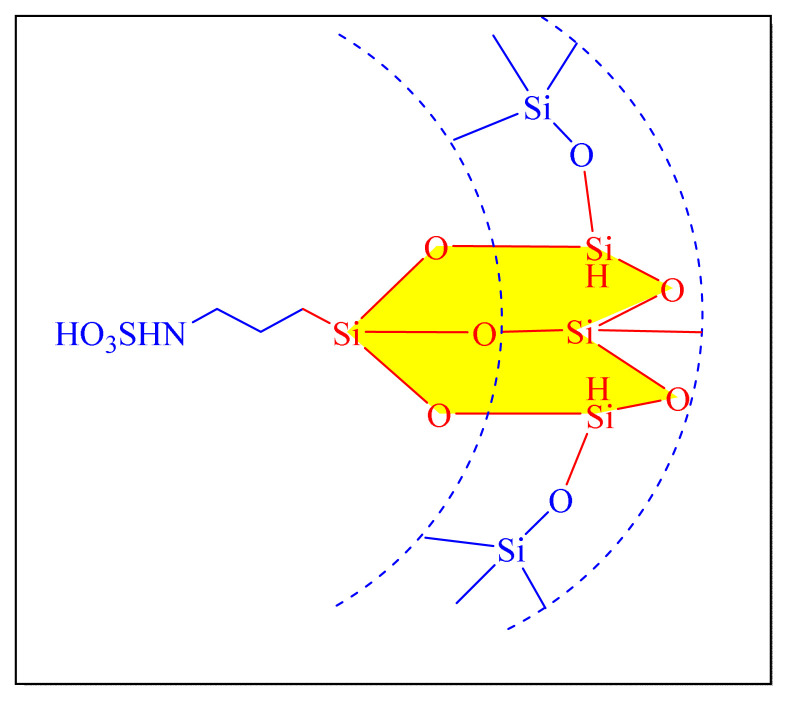
Suggested structure for nano-SnCl_4-_SiO_2_.

In 2013, Mirjalili et al. reported nanosilica support to stannic chloride [nano-SnCl_4_@SiO_2_, Figure 17, Table 6, Entry 11] which was prepared through a reaction of nanosilica oxide with stannic chloride. Nano-SnCl_4_@SiO_2_ was then used in the synthesis of 2,4,5-trisubstituted imidazole compounds in the absence of a solvent at 130 °C. This catalyst does not require any particular precautions for preparation, handling, or storing. It can be kept for months at room temperature without decreasing its catalytic performance [82].

**Figure 18:**
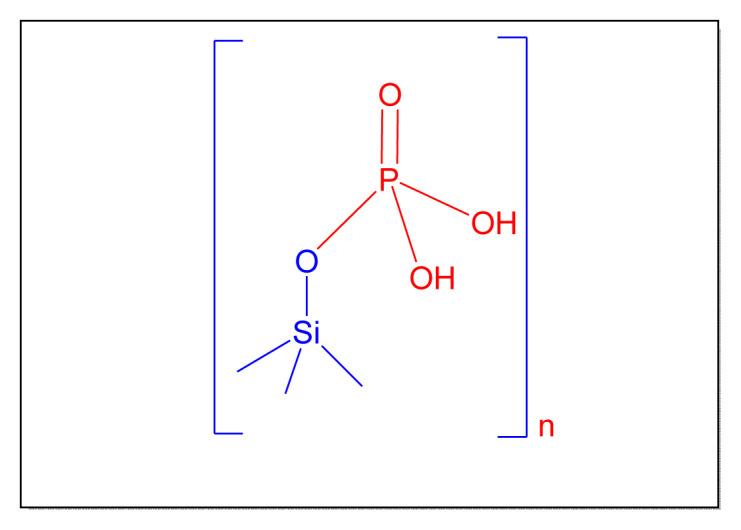
Nanosilica phosphoric acid (nano-SPA) catalyst.

Bamoniri et al. in 2012 described a simple method in order to synthesize 2,4,5-trisubstituted as well as 1,2,4,5-tetrasubstitued imidazoles derivatives by employing the nanosilica phosphoric acid catalyst [nano-SPA, Figure 18 Table 6, Entry 12] in the absence of a solvent in 2014. The retrieved catalysts were reused for three additional cycles, with each rerun resulting in progressive decrease in activity [83,84].

**Figure 19:**
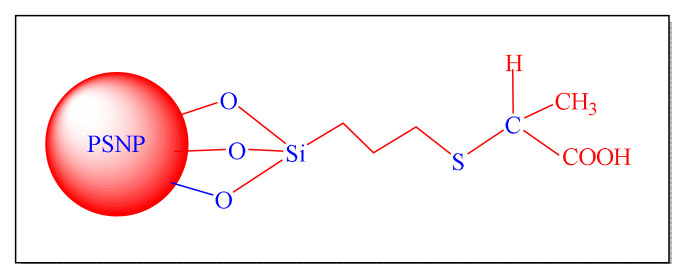
PSNP-CA nanocatalyst.

Ray et al. in 2013 have demonstrated chemoselective synthesis of the 2,4,5-trisubstituted as well as 1,2,4,5-tetrasubstitued imidazole compounds by employing a novel organometallic catalyst is carboxylic group functionalized porous silica nanoparticle [PSNP-CA, Figure 19, Table 6, Entry 13] in water at room temperature. The technique has three noteworthy features: first, the size of the silica nanoparticle can be detained among 20–25 nm with a very high surface to volume ratio; second, PSNP-CA allows “sensitive substances” to react under experimental parameters; and third, no trisubstituted imidazole is formed in the reaction, providing an extremely high selectivity for tetrasubstituted imidazole. In this approach, highlight a valuable advancement to the imidazoles synthesis. Although the protocol seems conventional, it is less impressive due to the use of a toxic solvent (toluene) during the preparation of catalyst and longer reaction times in more than three days required for the preparation of catalyst [85].

**Figure 20:**
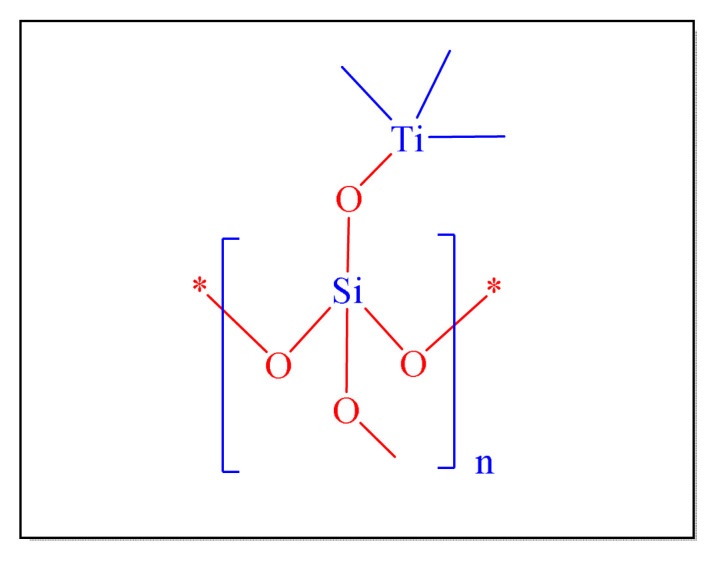
Suggested structure for nano-TiCl_4_-SiO_2_ catalyst.

In 2011, Mirjalili et al. performed a simple one-pot multicomponent approach in order to synthesis of 1, 2, 4, 5-tetrasubstituted imidazole compounds by employing 50% TiCl_4_-SiO_2_ as an efficient nanocatalyst [nano-TiCl_4_-SiO_2_, Figure 20 Table 6, Entry 14 ] under neat condition by conventional heating at 110 °C. Although there was a progressive drop in its potency, the catalyst may be reused [86].

**Figure 21:**
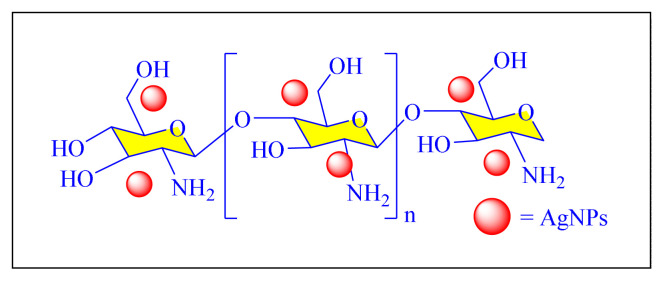
Structure of sliver nanoparticles on the surface of the chitosan.

In 2018, Hamzavi et al. employed efficient and green silver nanoparticles supported on chitosan [Ag NPs-Cs, Figure 21, Table 7, Entry 1] for the one-pot multicomponent synthesis of 2,4,5-trisubstituted as well as 1,2,4,5-tetrasubstitued imidazole derivatives in a neat condition by conventional heating. The solvent-free conditions, eco-friendly bionanocatalyst has made a significant contribution to reducing pollution formation; decreased use of hazardous and costly organic solvent, as well as simple operation, have emphasized the significant advantages of this method. The recovered catalyst’s reusability was verified for four consecutive cycles with nearly constant catalytic performance [87].

Shaabani et al. in 2017 have validated novel robust bionanoreactor catalyst that are crosslink chitosan (CS) nanosized particles anchored magnetically multiwall carbon nanotube [CS NPs/MWCNT@Fe_3_O_4_, Table 7, Entry 2] through ionotropic gelation method for the synthesis of the 2,4,5-trisubstituted imidazole derivatives. The present methodology found a new form of bionanoreactor catalyst, simple procedure, magnetically easily recoverable nanoparticles catalysts, simple set-up process, outstanding yields with shorter reaction durations and biocompatibility make the method beneficial over conventional approaches. Moreover, the bionanocatalyst showed a remarkable thermal stability at the desired temperatures, whereas, CS NPs/MWCNT@Fe_3_O_4_ nanocatalyst are more stable than Cu-CS NPs/MWCNT@Fe_3_O_4_ at high temperatures [88].

Shaabani et al. in 2017 have employed for the first time molecular imprinted polymers [MIP, Table 7, Entry 3] as nanoreactors prepared through mini-emulsion polymerization. They were used in the synthesis of 2,4,5-trisubstituted imidazoles compounds in absence of a solvent. Its greener and has a simple catalytic pathway, and the products are easily separated without the need for time-consuming purification methods like aqueous workup or chromatography. Furthermore, the catalyst was reusable for four times without significant loss of catalytic activity, and MIP morphology remained stable after the fourth attempt [89].

Singh and Rajput in 2017 have accomplished novel immobilization of Co(II) ions on to crosslinked chitosan–glutaraldehyde coated magnetic nanoparticles [MCS-GT@Co(II), Table 7, Entry 4] as nanocatalysts for the one-pot multicomponent condensation synthesis of 2,4,5-tri and 1,2,4,5-tetrasubstituted imidazole derivatives under reflux conditions in ethanol as a solvent. Four successive rounds of reusability were indicated, with almost no decrease in catalytic performance [90].

In 2016, Maleki et al. explored a green novel approach by employing cellulose supported ferric oxide and silver salt resulting nanocomposite as a nanocatalyst [cellulose/γ-Fe_2_O_3_/Ag, Table 7, Entry 5] which was employed for the synthesis of 2,4,5- trisubstituted imidazole derivatives in neat condition at 100 °C. The catalyst easily separated from the reaction mixture due to the excellent magnetic characteristics of nanocomposites. The retrieved catalyst successfully utilized five times in succession with no appreciable loss of catalytic activity [91].

**Figure 22:**
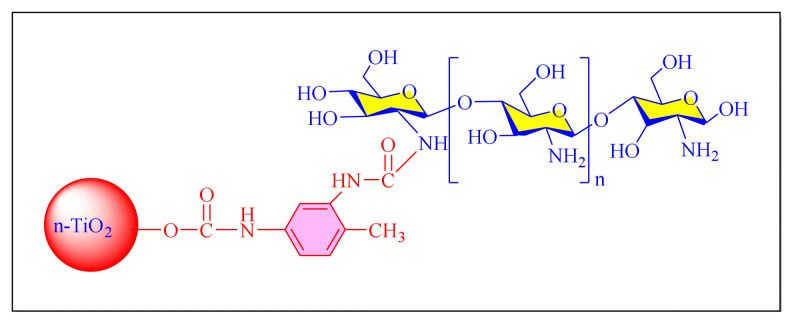
Nano-TiO_2_(n-TiO_2_@TDI@Chitosan) catalyst.

Ghasemi et al. in 2016 have applied novel chitosan-functionalized nanotitanium dioxide as a novel type of retrievable basic organocatalyst via covalently supported of 2,4-toluene diisocyanate [nano-TiO_2_(n-TiO_2_@TDI@Chitosan, Figure 22 Table 7, Entry 6] in order to synthesize 2,4,5-trisubstituted imidazoles under neat conditions at 100 °C. The prepared nanocatalyst characterized and found to be a robust and highly active heterogeneous nanocatalyst. Although the protocol seems conventional it is less impressive due to use of a toxic solvent (toluene) during catalyst preparation and longer reaction times (more than three days) required for the preparation of catalyst [92].

In 2015, Maleki et al. made a biocompatibility heterogeneous nanocatalyst composed of graphene oxide (GO)-chitosan nanocomposites [GO–chitosan, Table 7, Entry 7] It was used in the synthesis of 2,4,5-trisubstituted imidazole derivatives under neat conditions at 120 °C. Furthermore, the reaction has great conversion efficiency, a high yield, and is ecologically friendly. The recoverable GO–chitosan biodegradable nanocatalyst was reused six times in subsequent runs without a significant loss of catalytic activity [93].

Another green approach reported in 2015 by Girish et al. They have investigated one-pot multicomponent synthesis to prepared 2,4,5-trisubstituted imidazole derivatives by using Zirconium dioxide (ZrO_2_) supported β-cyclodextrin as nanocatalysts. [ ZrO_2_-β-CD, Table 7, Entry 8] They have the 1–5 nm range easily make by a simple one-pot coprecipitation process by using ZrOCl_2_.8H_2_O, and NH_4_OH. The retrieval catalyst used for four successive cycle, with every rerun resulting in a significant loss of activity [94].

In 2014, Zarnegar and Safari demonstrated an environmentally benign approach to synthesize 2,4,5-trisubstituted imidazole derivatives employing chitosan(CS)-coated ferrous ferric oxide(Fe_3_O_4_) as nanocatalyst [Fe_3_O_4_@CS, Table 7, Entry 9]. Fe_3_O_4_@CS nanocatalyst was prepared simply via coprecipitation method of Fe^2+^/Fe^3+^ ions and NH_4_OH in an aqueous medium of chitosan. The catalyst’s reusability was reported for six consecutive rounds, with a significant decrease in activity after each cycle [95].

In 2021, Sinha et al. have applied an environmental friendly novel aqueous phase protocol for the synthesis of trisubstituted imidazole compounds by employing Au supported on graphene oxide sheets as nanocatalyst [Au@RGO, Table 5, Entry 10] at 52 °C. It has gained a lot of interest for organic reactions because of its special advantages as an ecologically friendly and cost-effective solvent. Au@RGO nanocatalyst that appears to be cost-effective with recycled and reused seven times without losing catalytic performance in subsequent reactions [96].

Nayak et al. in 2020 have also applied a green and bio-based approach for the one-pot multicomponent condensation synthesis of imidazole compounds by using plant assisted tin oxide nanoparticles from the leaves of Ceropegia jainii plant as a nanocatalyst [Table 7, Entry 11] under microwave assistance with solvent-free circumstances. This approach is more efficient than previously reported methods because it has several advantageous aspects such as excellent yield and purity, microwave assistance, short reaction duration, no any side products, and solvent-free conditions [97].

In 2020, Kumar et al. introduced a novel and highly effective nanocatalyst [rGO-NiO-NCs, Table7, Entry 12] built on nickel oxide nanostructured materials deposited on a surface-functionalized reduced graphene oxide sheet via spontaneous air oxidation. The rGO-NiONCs was then used in the synthesis of 2,4,5-trisubstituted imidazole compounds in ethanol as a green solvent at 55 °C. Reusability of eco-friendly nanocatalyst was also shown without losing any activity even after five reaction cycle [98].

In 2020, Hebishy et al. reported another methodology for one-pot multicomponent synthesis of polysubstituted imidazoles by employing zinc oxide [ZnO, Table 7, Entry 13] heterogeneous nanocatalyst via reflux in methanol or acetic acid through the classical heating as well as microwaves assistant. The approach comprises rich library of imidazole scaffolds considered as a new lead compounds for selective antitumor agents against diverse cancer cells [99].

**Scheme 10:**
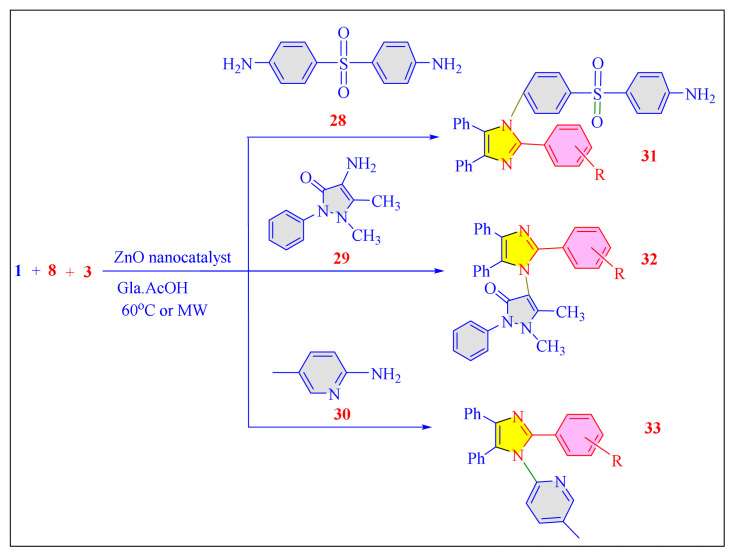
Synthesis of substituted imidazoles compounds by using ZnO nanocatalyst.

Jayashree et al. in 2019 have investigated an efficient, simple protocol for a new series of tetrasubstituted imidazole derivatives synthesized through one-pot multicomponent condensation reactions (MCRS). In this technique, reaction of 1,2-diphenylethane-1,2-dione (PhCO)_2_, benzaldehyde with substituents, a variety of amine scaffolds, or ammonium salt of acetic acid were achieved in glacial acetic acid by using zinc oxide nanoparticles as an effective nanocatalyst [Scheme 10, Table 7, Entry 14]. In this protocol catalyst activity performed under both the microwave (500W) and thermal conditions. Microwave-assisted protocol proved most efficient with shorter reaction times and higher yields. All synthesized products screened for antibacterial activity found high potential over the selected bacterial strain and thus a promising extension to the existing antibacterial agents to make the valuable contribution. The catalyst systems were observed to be recycled for three subsequent cycles in a row, with a significant loss of activity it after each cycle [100].

In 2018, Zahedi et al. achieved eco-friendly and effective one-pot multicomponent condensation reaction to the synthesize substituted imidazoles through employing the silica-supported La0.5 Pb0.5MnO_3_ green solid acid perovskite-type nanoparticles [S-LPMO nanoperovskite, Table 7, Entry 15] with reflux in ethanol and under the solvent-free condition. The ethyl alcoholic reaction mixture containing substituted imidazole components were refluxed in the presence of S-LPMO nanocatalyst afforded good amounts of products over to solvent-free condition. The catalyst used in this technique has a larger specific surface area, which increases the degree of interaction between the catalyst and the reactants. This approach has various advantages, including high yields, fast reaction durations, ease of operation, and facile separation and recyclability of the catalyst [101].

In 2016, Allahvirdinesbat et al. have applied H–ZSM-5 supported nanosized bimetallic oxide nanoparticles [Ag–Fe/ZSM-5, Table 7, Entry 16] exhibiting excellent catalytic activity to synthesize tri/tetrasubstituted imidazole derivatives under neat conditions. The protocol has several advantages such as atom economic green approach, shorter reaction duration, ease of operation and facile to separation. Polar solvents and neat conditions afforded better yield than nonpolar. It was reported that the system could be reused for four more cycles with a minor drop in activity after each rerun [102].

Maryam et al. in 2014 have validated nano-TiO_2_ supported on SiO_2_ solid acid catalyst [TiO_2_@SiO_2_, Table 7, Entry 17] which was employed for the synthesis of 2,4,5- trisubstituted as well as 1,2,4,5-tetrasubstituted imidazoles in methanol as solvent at room temperature. Furthermore, they were able to perform the reaction at using a small quantity of the catalyst. The catalyst’s reusability decreases after five cycles with very minimal activity [103].

In 2014, another environment friendly approach reported by Safa et al. They have attempted a novel method and highly efficient one-pot multicomponent condensation reactions to synthesize 1,2,4,5-tetrasubtituted imidazole derivatives by employing the ZSM-5 supported bimetallic oxide [ Fe–Cu/ZSM-5, Table 7, Entry 18] as a nanocatalyst under solvent-free conditions. This approach provides a very quicker and minimal cost process for the preparation of imidazoles products and also Fe–Cu/ZSM-5 catalyst known as heterogeneous “E” catalyst (efficient, eco-friendly and economic). Furthermore, no side products such like trisubstituted imidazole compounds, oxidative aniline products, or aldehydes, which are commonly found when strong acids are used, were detected in this technique [104].

Borhade et al. in 2012 have reported a novel green and highly efficient SiO_2_ supported SnO_2_ [SiO_2_-SnO_2_, Table 7, Entry 19] nanocatalyst for the one-pot multicomponent synthesis of tri/tetra-tetrasubstituted imidazoles at 80 °C. The average size of the SiO_2_:SnO_2_ nanocrystallite found in rang about 62.3-nm. It has received more attention because of its excellent thermal stability, vast surface area, ease of recover, and ability to carry out reactions at lower temperatures. The catalyst’s reusability was demonstrated for five additional cycles with no significant decrease in catalytic activity [105].

In 2018, Bamoniri et al. introduced a new type of solid acid nanocatalyst, which is constructed based on reaction of nanomaterial of kaolin supported with chlorosulfonic acid [nano kaolin-SO_3_H, Table 8, Entry 1]. It was applied for the one-pot multicomponent synthesize of trisubstituted imidazole compounds in the neat condition. Authors reported high yields, simple reaction conditions, and short reaction times as significances of the method [106].

Kolvari et al. in 2016 have attempted eco-friendly synthesis tri- and tetrasubstituted imidazoles under solvent-free conditions by employing nanoceramic Tile Waste-SO_3_H (n-CTW-SA) as a catalyst. They made nanocatalyst out of the waste ceramic tiles which then supported by sulfonic acids [Table 8, Entry 2]. The catalyst systems utilized on waste products and afforded high to excellent yields. The recovered catalyst was reused for seven runs under the identical conditions with no discernible decrease in yield or catalytic performance [107].

In 2015, Kolvari et al. have synthesised tri- and tetrasubstituted imidazole derivatives through the one-pot multicomponent reaction under the solvent-free conditions by using perlite supported fluoroboric acid as a nanocatalyst [n-PeFBA, Table 8, Entry3]. The method showed significances such as use of natural catalyst support, economic yields and efficient reusability [108].

Safa et al. in 2015 have developed a novel environmentally benign one-pot multicomponent synthesized of 1,2,4,5-tetrasubstituted imidazole derivatives by using Cu/SAPO-34 zeolite nanocatalyst [Table 8, Entry 4 ]. The use of water as a green solvent under ultrasonic conditions, excellent yields makes the protocol notable [109].

Alinezhad et al. in 2018 have performed eco-friendly synthesis of one-pot synthesis of 2,4,5-tri as well as 1,2,4,5-tetrasubstituted imidazoles derivatives by using acid ionic liquids [H-NP]HSO_4_ as a new and high effective nanocatalyst [ [H-NP]HSO_4_ (H-NPBS), Table 8, Entry 5] under neat conditions. Low vapour pressure, strong thermal stability, high ion conductivity, and easy functionality are all unique features of the [H-NP]HSO_4_ (H-NPBS) catalyst. The nanocatalyst was reused six times in succession, with a modest drop in efficiency after each rerun [110].

Vaghei et al. in 2018 have proposed a novel and efficient approach for the one-pot multicomponent synthesis of the 2,4,5-trisubstituted and 1,2,4,5-tetrasubstituted imidazole derivatives by using the H_3_PW_12_O_40_/Fe_3_O_4_@SiO_2_–Pr–Pi nanocatalyst [Table 8, Entry 6] in ethanol under reflux conditions. In this protocol, efficiency demonstrated by its high product yield and separation of the catalyst with external magnetic field is the notable advantage. In addition, the antifungal property of the imidazole derivatives was assessed by the cultivation of the fungus *Fusarium oxysporum* on potato dextrose agar medium containing them. This methodology is less impressive due to toxic solvent (dry toluene) used during the preparation of catalyst [111].

**Figure 23:**
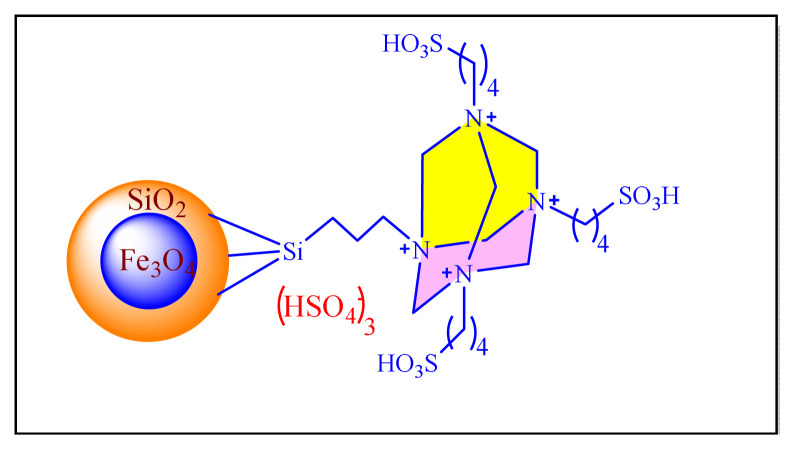
Fe_3_O_4_@SiO_2_-HM-SO_3_H magnetic nanocatalyst.

Another green approach in 2015 have reported by Naeimi et al. is introduced an effective nanocatalyst Fe_3_O_4_@SiO_2_.HM.SO_3_H for the one-pot multicomponent synthesis of 2,4,5-trisubstituted imidazole derivatives under solvent-free microwave irradiated conditions [Figure 23,Table 8, Entry 7]. Catalyst showed excellent catalytic activity, microwave-assisted and a solvent-free conditions are all key advantages of the suggested methodology [112].

**Figure 24:**
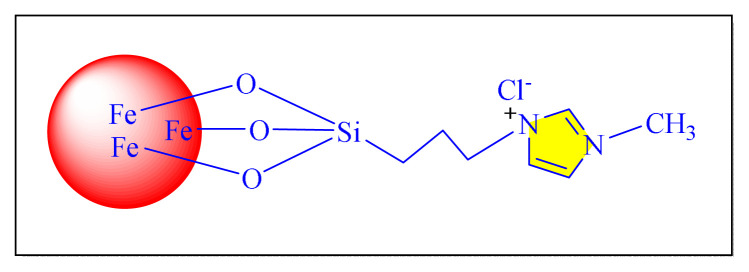
IL-MNPs catalyst.

In 2013, Safari et al. validated a green and effective one-pot multicomponent sonochemical synthesis of tetrasubstituted imidazole derivatives using a super paramagnetic ionic liquid nanocatalyst [IL-MNPs, Figure 24, Table 8, Entry 8] in the presence of ethyl alcohol at room temperature. The current synthetic approach was demonstrated under solvent-free microwave-assisted as well as conventional heating conditions. The supported catalysts quickly isolated from the reaction mixture, magnetically. The IL-MNPs catalyst was reused for five cycles in a row, with a slightly decline activity after each rerun [113, 114].

In 2019, Khalifeh et al. have applied copper nanoparticles supported over charcoal (Cu/C) as an excellent catalyst [Table 8, Entry 9] for the one-pot, three-component synthesis of 2,4,5-trisubstituted imidazole compounds in PEG 200 is used as an eco-friendly solvent at 100 °C. In this technique various solvents were utilized, however it was observed that PEG 200 offered the best yield of product at very short reaction times, as well as lower temperatures. This method offers some key features such as use of readily available starting materials, easy recovery of catalyst and catalyst reusability [115].

**Scheme 11:**
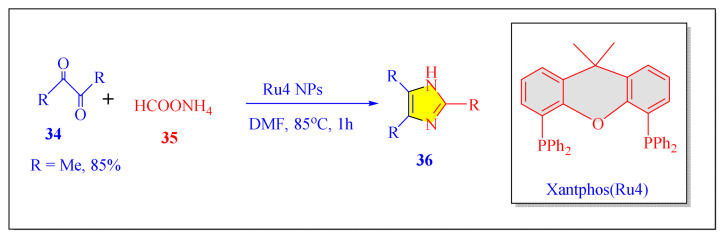
Synthesis of trisubstituted imidazoles derivatives.

In 2017, Ganji et al. have demonstrated a novel synthetic protocol for the synthesis of 2,4,5-trisubstituted imidazole compounds by employing the phosphine (Xantphos) support with ruthenium nanoparticles (RuNPs) as catalysts [Scheme 11,Table 8, Entry 10] in presence of DMF at 85 °C. The method utilized α-β diketones for the synthesis of imidazoles makes it remarkable. Furthermore, the catalyst are easily removed in reaction mixture through the adsorption on silica or alumina [116].

**Figure 25:**
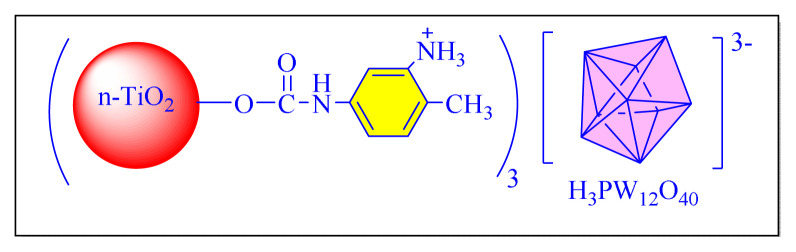
n-TiO_2_–NH_2_/HPW nanocatalyst.

In 2017, Shamsi et al. evaluated a new class of zwitter-ionic heterogeneous nanocatalyst [n-TiO_2_–NH_2_/HPW, Figure 25, Table 8, Entry 11] applied for the one-pot multicomponent synthesis of 2,4,5-trisubstituted imidazole compounds under neat conditions at 97 °C. After each rerun, the recovered catalyst showed a modest drop in activity. The use of a hazardous solvent (dry toluene) during catalyst synthesis limits the credit of method [117].

## 4. Organic polymers

**Figure 26:**
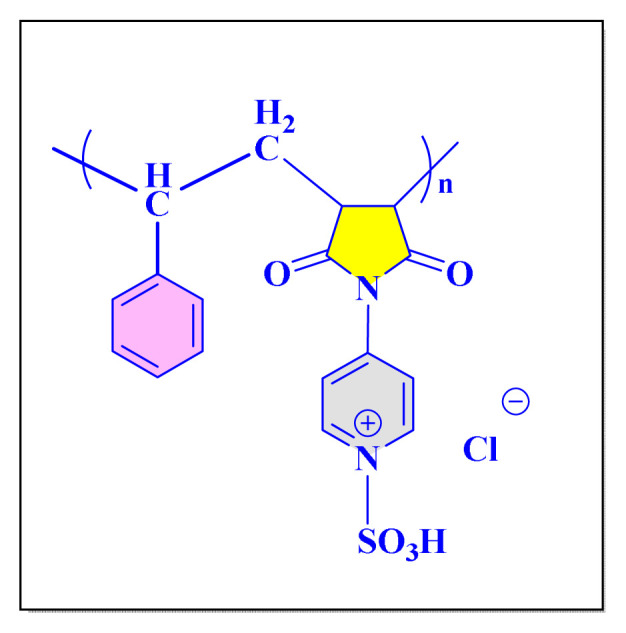
SMI-SO_3_H catalyst.

In 2014, Heravi et al. have reported novel solid acid SMI-SO_3_H catalyst for the green synthesis of 1,2,4,5-tetrasubstituted imidazole derivatives under neat conditions at 90 °C [Figure 26, Table 9, Entry 1]. The eco-friendly, commercially available copolymer polystyrene-co-maleic anhydride (SMA) was supported on chlorosulfonic acid. The thermal stability of catalyst, cost-effective technique with high yields, recovery, and reusability of metal-free solid acid heterogeneous catalyst found noticeable features of this protocol [118].

**Figure 27:**
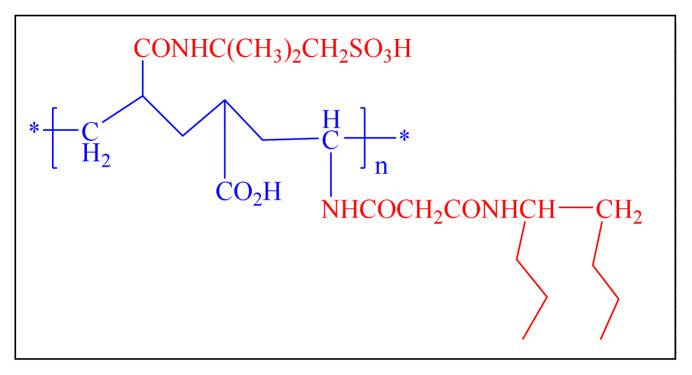
Crosslinked polymeric [poly (AMPS-co-AA)] catalyst.

Mohammadi et al. in 2011 have presented efficient and eco-friendly approach for the one-pot multicomponent synthesis of tri/tetrasubstituted imidazole compounds is achieved by crosslinked polymer catalysts [poly(AMPS-co-AA), Figure 27, Table 9, Entry 2] under solvent-free conditions. Furthermore, the reactivity pattern is exceedingly cleanly with no side products, products purifying using nonchromatographic methods and also great catalyst reusability [119,120].

**Figure 28:**
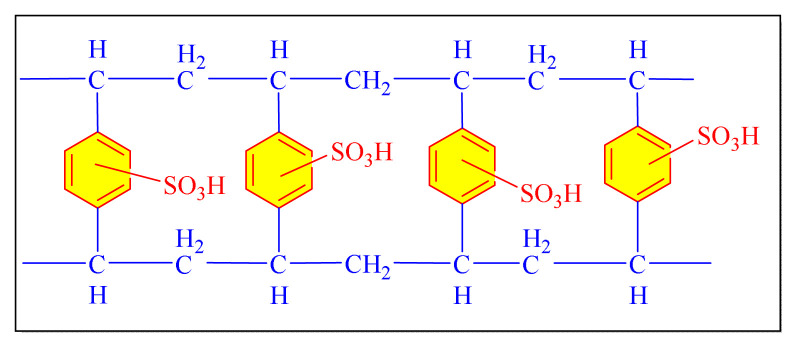
Amberlite IR 120H^+^ resin.

In 2017, Chakraborty et al. introduced highly efficient Amberlite IR 120H^+^ resin is a gel-type cross linking polymer bearing –SO_3_H group in the exterior as a catalyst [Amberlite IR 120H^+^, Figure 28 Table 9, Entry 3] for synthesis of tri- and tetrasubstituted imidazole derivatives under ethanol at 80 °C. Simple work up procedures, high economy and an efficient reusability of catalyst makes method notable [121].

Pandit et al. in 2011 have discussed here simple and green approach for the one-pot multicomponent condensations for the synthesis of tri- and tetrasubstituted-1H-imidazole derivatives by employing Amberlyst A-15 catalyst [Table 9, Entry 4] under solvent-free conditions at MW irradiation. The catalyst performed well under mild conditions; provided high yields [122].

Penumati et al. have reported an efficient method for the synthesis of multisubstituted imidazoles by using poly-ethylene glycol [PEG-400, Table 9, Entry 5] as green reaction conditions. PEG-400 serves as both a phase transfer catalyst and a clean solvent. PEG and its derivatives become more popular as an alternate reaction media, due to their interesting properties like nontoxicity, biocompatibility, and biodegradability makes the protocol remarkable [123].

Kumar et al. in 2014 have proposed efficient and eco-friendly synthesis of 2,4,5-trisubstituted-1H-imidazole compounds by using PEG-400 as phase-transfer heterogeneous catalysts [PTCs, Table 9, Entry 6] in glacial acidic acid. Simple work up procedures, high yields and purification by recrystallization seems notable [124].

In 2010, Nalage et al. demonstrated without catalyst efficient and green process to the synthesis of 2,4,5-trisubstituted imidazole compounds under PEG as solvent [Table 9, Entry 7] through microwave assistant for 5 to 10 min at 900W. The method performed under different solvents like PEG-200, 400, 600, but no difference in yield observed. This protocol, employed greener approach but provided average yields of the products [125].

In 2010, Hoseini et al. reported carbon-based solid acids as a heterogeneous catalyst [Table 9, Entry 8] for the synthesis of 1,2,4,5-tetrasubstituted imidazole derivatives under neat conditions at 130 °C for 1–2 h. The catalyst was prepared by heating aromatic compounds such as naphthalene in sulphuric acid. After each rerun, the reusability of retrieved catalyst was mentioned for successive cycles with no noticeable reduction in activity [126].

## 5. Hybrid polymers

In 2018, Gabla et al. synthesized solid acid heterogeneous catalyst like the propyl-SO_3_H group supported on Santa Barbara Amorphous-15 [SBA-15–Pr– SO_3_H, Table 10 Entry 1] via the one-pot condensation method. The catalyst was applied for the green and rapid synthesis of trisubstituted and tetrasubstituted imidazole compounds under neat conditions and microwave irradiation. The recovered catalyst was reused for the five times with a slight decrease in activities after each cycle [127].

Gorsd et al. in 2016 have attempted the catalytic activity of a PS@SiTPA30 for the one-pot multicomponent synthesis of 2,4,5-trisubstituted imidazole compounds in absence of solvent [PS@SiTPA30, Table 10, Entry 2]. A catalyst has been prepared by supporting tungstophosphoric acid on core-shell polystyrene-silica microspheres or hollow silica spheres. This approach is notable for its excellent selectivity and lack of side products. The retrieved catalysts has been reused three times in a row, with a slightly decrease in activity after each rerun [128].

**Figure 29:**
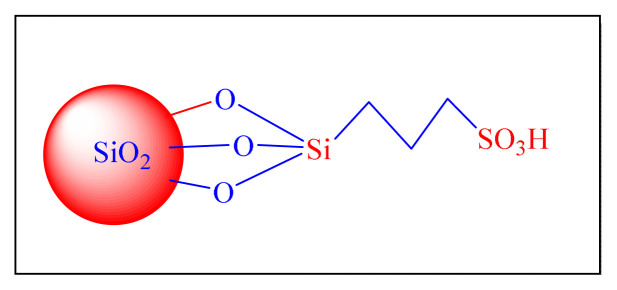
SiO_2_-Pr-SO_3_H catalyst.

In 2013, G. Ziarani et al. have introduced a novel sulphonic acid support on SiO_2_ as an effective solid acid catalyst [SiO_2_-Pr-SO_3_H, Figure 29, Table 10, Entry 3]. The catalyst used for the synthesis of tetrasubstituted imidazole compounds through the one-pot multi component condensations reaction under the solvent-free conditions at 140 °C. In this protocol, the SiO_2_-Pr-SO_3_H catalyst was reused more than once without losing activity. The catalyst preparation involved use of toluene as solvent, which adds to the disadvantage of method [129].

In 2021, Manteghi et al. have reported a novel and effective chromium-containing metal-organic framework (MOF) [MIL-101 (Cr), Table 10, Entry 4] as a heterogeneous catalyst. The one-pot multicomponent synthesis of trisubstituted imidazole compounds was attempted using MIL-101 (Cr) catalyst under neat condition at 120 °C. The recovered catalyst was recycled five times in a succession with no discernible decrease in catalytic performance [130].

Ramezanalizadeh et al. in 2016 have prepared highly efficient cobalt/nickel bimetallic metal organic framework (MOF) catalyst from terephthalic acid and pyrazine [(Co/Ni)- (μ3-tp)_2_ (μ2-pyz)_2_ MOF, Table 10, Entry 5]. This bimetallic MOF applied for the one-pot multicomponent condensation synthesis of 2,4,5-trisubstituted imidazole compounds in absence of solvent at 120 °C. Eco-friendly reaction conditions and excellent catalytic activity add impression to this method. Although, the MOF catalyst proved itself as efficient but, its preparation and characterization seems costlier. The catalyst can be reused at least five times without losing significant catalytic activity [131].

**Figure 30:**
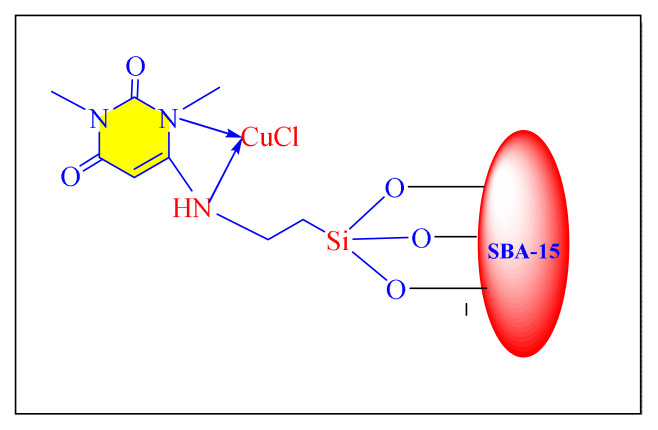
Copper (l) modified SBA-15 catalyst.

In 2017, Hajjami et al. have proposed simple and eco-friendly method for the synthesis tri/tetrasubstituted imidazole derivatives by employing Cu(I)-1,3-dimethylbarbituric acid modified SBA-15 as catalyst [Cu/SBA-15, Figure 30, Table 10, Entry 6] under the solvent-free condition at 100 °C. The reusability of retrieved catalyst was reported for five more runs with nearly constant catalytic activity. The method introduced a new type of hybrid catalyst, but it had some limitations because it required prolonged (almost nine days) catalyst preparation processes [132].

**Figure 31:**
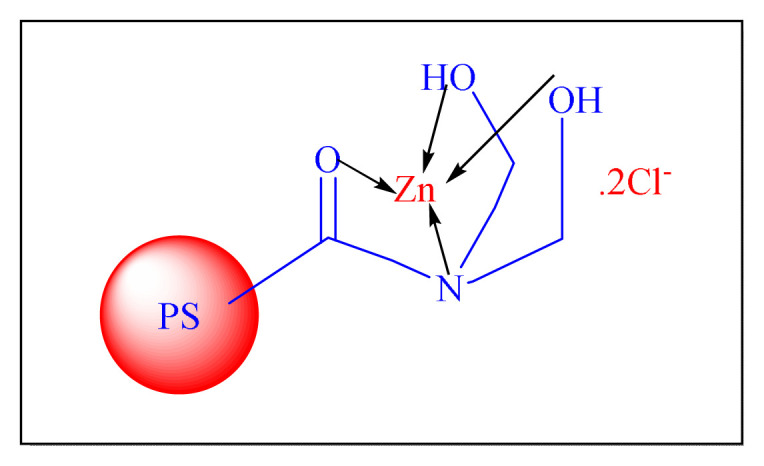
Polymer-supported zinc catalyst.

Wang et al. in 2008 have presented polymer-supported zinc catalyst [Table 10, Figure 31, Entry 7] prepared by chloracetylated polystyrene resin which first reacted with diethanolamine (DEA) and later loaded with ZnCl_2_. It was applied for the synthesis of 2,4,5-trisubstituted imidazole compounds under refluxing ethanol. The approach looks also unique because the immobilised catalyst is very stable, readily distinguishable from the reaction mixture, recyclability, higher selectivity, improved stability, and nontoxicity. The recovered catalyst was recycled four times in a row without noticeable decrease in catalytic performance [133].

## 6. Biocatalysts

In 2021, Londhe et al. demonstrated an ecologically sustainable technique for the one-pot multicomponent synthesis of tri-substituted imidazoles. They have employed activated dry baker’s yeast as a biocatalyst [Table 11, Entry 1] and the methanolic reaction mixture was subjected to ultrasonication at room temperature. Simple reaction conditions, use of nontoxic organic solvent, no side reactions and not generation of acidic/metallic wastes highlighted the impression of the method. Also product recovery from organic solvent is relatively easy, since enzymes are insoluble in organic solvents, allowing for facile recovery [134].

In 2020, Maleki et al. reported a novel green and efficient cellulose-pumice magnetic catalytic system [Table 11, Entry 2] for the synthesis of 2,4,5-triarylimidazole compounds in ethanol by ultrasonication. The useful advantages of this catalytic system include biodegradability, extremely high active surface area and simple separation of the catalyst. Moreover, using external magnetic field, the natural pumice was successfully retrieved. The recovered catalyst could be recycled ten times without losing its catalytic efficiency [135].

**Figure 32:**
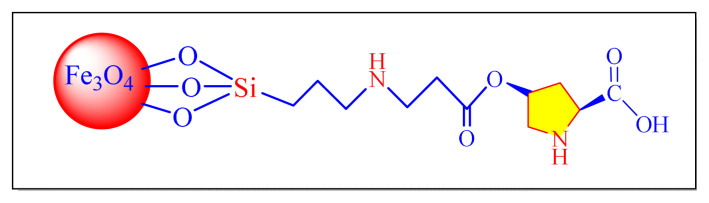
Magnetic OAc-HPro@Fe_3_O_4_ nanobiocatalyst.

In 2017, Aghahosseini et al. have reported innovative magnetic nanostructure-based L-proline as bionanocatalyst [OAc-HPro@Fe_3_O_4_, Figure 32, Table 11, Entry 3]. This magnetic bionanocatalyst was effectively used for the synthesis of 2,4,5-trisubstituted as well as 1,2,4,5-tetrasubstituted imidazole compounds under ethanol at 60 °C. The catalyst’s reusability for five successive rounds indicated, with low activity decrease. Despite the fact that, the process appears to be typical it takes lengthier reaction durations [136].

**Figure 33:**
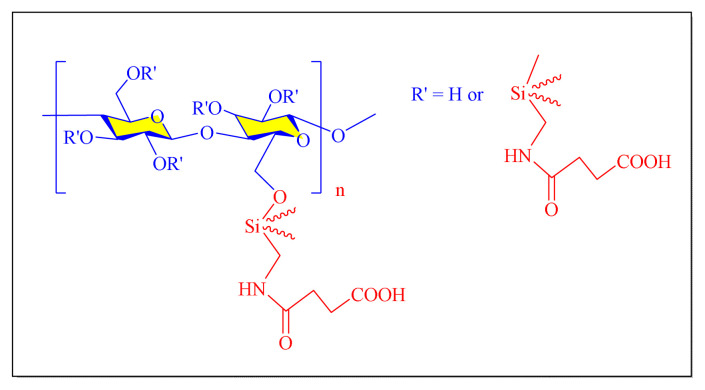
COPAPSC catalyst.

Salimi et al. in 2015 have introduced metal-free, green, solid supported biodegradable modified acid functionalized cellulose [COPAPSC, Figure 33 Table 11, Entry 4] as a biocatalyst for the solvent-free synthesis of 2,4,5-trisubstituted as well as 1,2,4,5-tetrasubstituted imidazoles. The retrieved catalyst can be recycled up to four times in consecutive reactions without losing considerable efficacy. This technology appears to be less impressive due to the use of a toxic solvent during catalyst preparation as well as prolonged reaction times [137].

**Figure 34:**
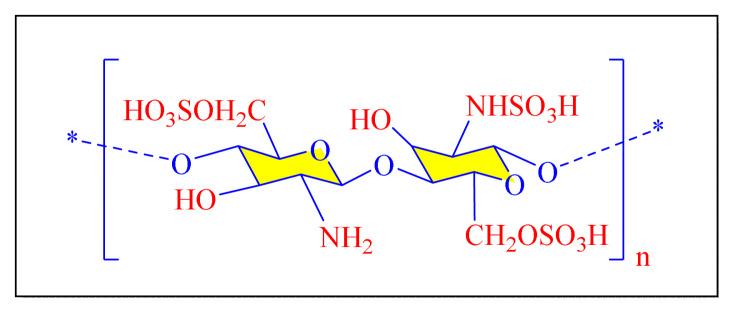
Chitosan-SO_3_H catalyst.

In 2015, Khan et al. have achieved eco-compatible, highly efficient one-pot multicomponent synthesis of tri/tetrasubstituted compounds by employing chitosan-SO_3_H biocatalyst [CTSA, Table 11, Figure 34 Entry 5,] under ethanol at microwave assistant. The present protocol several significant advantages such as environmental begins biocatalyst, chromatography-free purification, and elimination of environmentally hazardous solvents. This strategy appears to be with lengthier reaction times. The retrieved catalyst was reused five times in succession, with each rerun resulting in a significant loss of activity [138].

An environmental friendly protocol reported in 2012 by Zheng et al. which involves the application of lipase AT30 biocatalyst for the synthesis of 2,4,5-trisubstituted imidazole derivatives [Table 11, Entry 6] in ethanol. The use of a lipase enzymatic catalyst in this approach seems attractive for promoting the cyclization process and obtaining high yields of imidazole derivatives. *Lipase* found more attractive and effective catalyst than *esterase* as it accelerates the dehydration process. Due to the lack of reusability of the catalyst and the longer reaction time, the protocol restricts the credit [139].

Ramesh et al. in 2011 validated facile and efficient one-pot synthesis of 2,4,5-trisubstituted and 1,2,4,5-tetrasubstituted imidazole derivatives by employing readily available bioglycerol-supported carbon solid acid as a catalyst [Table 11, Entry 7] under acetonitrile. Moreover, the said bioglycerol-based acid catalyst can easily be prepared in the laboratory and is economically viable since the starting material is readily available [140].

In 2009, the group of Shelke have explored bio-supported cellulose sulphuric acid as an environmentally benign catalyst [ CSA Table 11, Entry 8] for the one-pot multicomponent solvent-free microwave-assisted synthesis of 2,4,5-triarylimidazole compounds. Furthermore, the catalyst can be reused up to four times without losing significant activity, making the method cost-effective [141].

**Figure 35:**
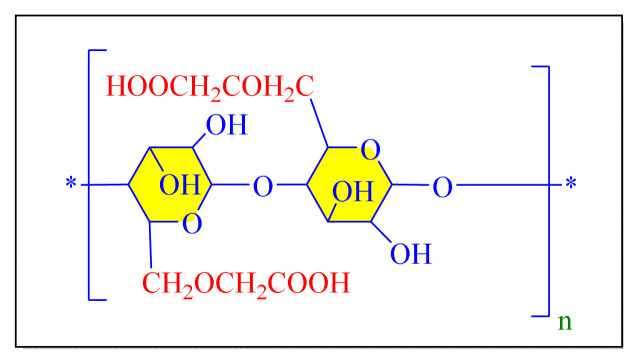
Structure of CMC.

Wang et al. in 2009 have introduced polymer-supported rare-earth lanthanide catalysts with carboxymethyl cellulose (CMC) [ Ln-CMC, Figure 35, Table 11, Entry 9] for the one-pot multicomponent synthesis of 2,4,5-trisubstituted imidazole derivatives in THF. The Ln-CMC catalyst showed higher catalytic activity than other mentioned lanthanide catalysts. The catalyst is stable and storable in air and can be easily prepared in one-step from commercially available CMC. However, the protocol appears to limit credit by requiring a longer reaction time [142].

## 6. Conclusion and future perspectives

This review article summarises the most recent work mainly on role of polymers as a reagent support, catalyst support, or as a catalyst. The present perspective will facilitate the creation of more efficient one-pot procedures for the synthesis of different multisubstituted imidazole compounds, which have been employed. Over hundred articles demonstrating significant advancements in recent decades for preparations of imidazoles were critically reviewed. Tremendous efforts were contributed to the amazing improvement and progress in imidazoles synthesis over the traditional or conventional methodologies. The progressions in the imidazole reaction, which are impressively worth mentioning, include simple heterogeneous set-up procedure, recyclability of polymers catalyst, ultrasonic irradiation, microwave assisted, and solvent-free conditions, which reported higher yields of desired multisubstituted imidazole compounds by green methods such as using a green polymer or solid supported catalyst. Microwave-assisted reactions found comparatively attractive due to features such as eco-friendly, atom-economical procedures, higher yield, lesser reaction times, regio- and chemoselectivity in the imidazoles synthesis.

Because of their outstanding catalytic activity, excellent stability, and selectivity, the fact that a variety of polymer or solid supported catalyst components could be constructed using simplified procedures from readily available and inexpensive starting materials, broadens the concept of their applications. Polymers in organic, inorganic, hybrid, nano, and biocatalyst forms have been also described as a heterogeneous catalyst with high catalytic activity and reusability. In the last decade, nanocatalysis has contributed significantly to the improvement of the conventional multisubstituted imidazole reaction with recovery and recycling of catalysts, specifically magnetic nanocatalysts, and simplified work-up techniques. Nanocatalysis has proven to be a new and effective technology; although the synthesis and characterisation of nanocatalysts appears to be more expensive than that of other heterogeneous catalysts. This may limit the valuable impression of catalytic advancement. In this scenario, researchers challenged to develop polymeric catalysts support that can perform under modest, lower reaction temperature conditions with optimal output and with reusability. We hope that this literature will encourage additional innovative research in a variety of disciplinary fields, as well as provide useful information on how to discover and deploy new kinds of polymer-supported catalysts for future research challenges.

## Figures and Tables

**Scheme 9:**
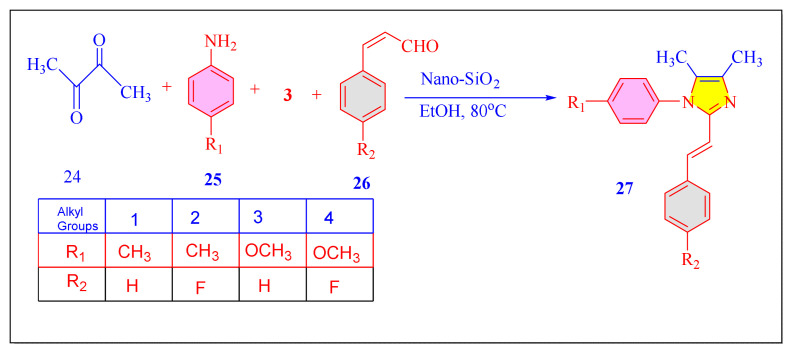
Synthesis routs of imidazoles derivatives.

**Table 1:** Classical routes for the synthesis of imidazoles.

1. Debus synthesis	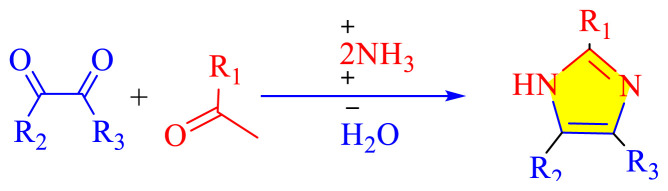
2. Radiszewski synthesis	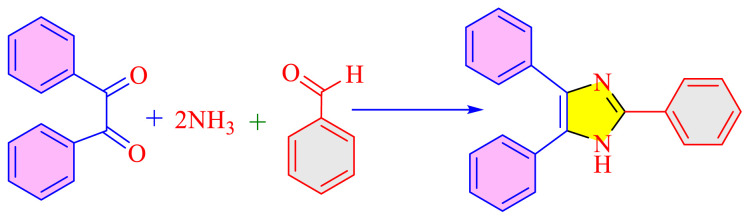
3. Dehydrogenation of imidazolines	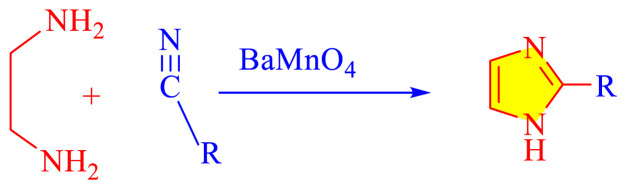
4. Imidazole from alpha halo ketones	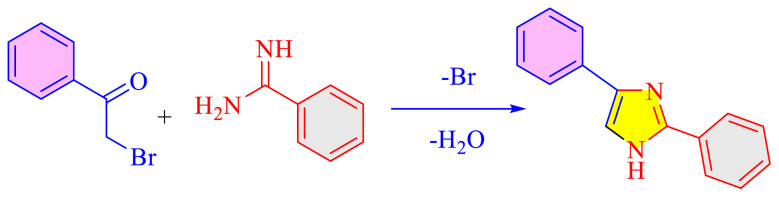
5. Wallach synthesis	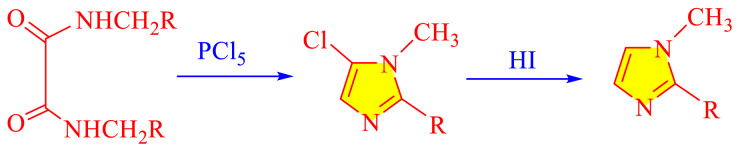
6. Marckwal synthesis	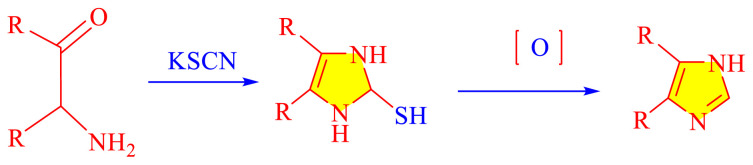

**Table 2:** Silica based polymeric catalysts for synthesis of imidazole derivatives.

Types of inorganic solid or polymer catalysts	Entry	Polymer support catalysts	Quantity of catalyst (^a^g,^b^mol %,^c^ wt %)	Solvent conditions	Energy medium	Temp. in °C (rt = room temp.)	Time (^d^ h, ^e^ min, ^f^ s)	Yield (%)
**Silica**	1	TiCl_3_- SiO_2_ (2020)	1.0^a^	-	Heat	90 °C	4^d^	92
	2	SiO_2-_Cu_2_O (2015)	0.2^a^	Ethanol	Heat	80 °C	15^e^	90
	3	SbCl_3_-SiO_2_ (2014)	0.1^a^	-	MW	-	10^e^	95
	4	SiO_2_–Cl (2013)	0.25^a^	-	Heat	80 °C	30^e^	80
	5	SBA-15 (2012)	0.1^a^	TFE	Heat	90 °C	3^d^	82
	6	H_2_SO_4_-SiO_2_ (2012)	0.015^a^	-	Heat	110 °C	1^d^	90
	7	HBF_4_-SiO_2_ (2012)	0.04^a^	-	Heat	120 °C	15^e^	95
	8	P2O_5_-SiO_2_ (2011)	0.05^a^	-	Heat	100 °C	60^e^	98
	9	TiCl_4_-SiO_2_ (2011)	0.3^a^	-	Heat	120 °C	60^e^	90
				-	MW	-	10^e^	96
	10	SbCl_5_-SiO_2_ (2010)	0.15^a^	Ethanol	Heat	Reflux	45^e^	96
				-	Heat	140 °C	120^e^	96
	11	Zr-CAP-SG (2010)	10^c^	CH_3_CN	Heat	rt	4.45^e^	87
	12	SBSSA (2009)	0.002^a^	-	Heat	130 °C	30^e^	95
	13	MoO_3_-SiO_2_ (2009)	10^c^	CH_3_CN	Heat	80 °C	2.2^d^	95
	14	BF_3_.SiO_2_ (2008)	37^c^	-	Heat	140 °C	2^d^	80
	15	HClO_4_ –SiO_2_ (2006)	1^b^	-	Heat	140 °C	20^e^	96
	16	SSA (2006)	0.2^a^	-	Heat	130 °C	85^e^	92
					MW	-	10^e^	89
	17	NaHSO_4_ –SiO_2_ (2006)	1^a^	-	MW	-	6^e^	90
	18	Silica gel (2000)	2^a^	-	MW	-	6^a^	91

(Table 2 summarizes different silica based catalysts and covers the reaction conditions, catalyst amount and yields under which variety of catalysts investigated. Table 2 emphasized the information of individual catalyst only, comparison between catalysts cannot be considered due to diversely substituted reactants.)

**Table 3:** Clays and minerals based polymeric catalysts for synthesis of imidazole derivatives.

Types of inorganic solid or polymer catalysts	Entry	Polymer support catalysts	Quantity of catalyst (^a^g, ^b^mol %,^c^ wt %)	Solvent conditions	Energy medium	Temp. in °C . (rt = room temp.)	Time (^d^ h, ^e^ min, ^f^ s)	Yield (%)
**Clay and mineral**	1	Activated fuller’s earth (2021)	0.2^a^	-	Heat	100 °C	2^d^	95
	2	Zeolite ZSM-11 (2021)	0.05^a^	-	Heat	110 °C	30^e^	90
	3	Red Brick Clay Powder (2019)	0.02^a^	Water	Heat	60 °C	25^e^	96
	4	Ti^4+^/4 Å MS (2018)	0.1^a^	Toluene	Heat	100 °C	10^d^	80
	5	HPVAC-20 (2017)	0.05^a^	-	Heat	110 °C	25^e^	96
	6	Kaolin-SO_3_H (2014)	0.05^a^	-	Heat	110 °C	2.5^d^	91
	7	Fe(NO_3_)_3_-Kie (2013)	1.6^b^	-	Heat	120 °C	1^d^	86
	8	K10Ti (2013)	0.25^a^	-	Heat	120 °C	2^d^	80
	9	Cu(NO_3_)_2_-Zeolite (2010)	0.03^a^	-	Heat	80 °C	30^e^	96
	10	Scolecite (2009)	2^c^	Ethanol	Heat	Reflux	30^e^	95

(Table 3 summarizes different catalysts belonging to clay and mineral category and covers the reaction conditions, catalyst amount and yields under which variety of catalysts investigated. Table 3 emphasized the information of individual catalyst only, comparison between catalysts cannot be considered due to diversely substituted reactants.)

**Table 4:** Heteropolyacids and ions, PPA and alumina based polymeric catalysts for synthesis of imidazole derivatives.

Types of inorganic solid or polymer catalysts	Entry	Polymer support catalysts	Quantity of catalyst (^a^g,^b^mol %,^c^ wt %)	Solvent conditions	Energy medium	Temp. in °C . (rt = room temp.)	Time (^d^ h, ^e^ min, ^f^ s)	Yield (%)
**Hetero polyacid and ions**	1	Silica-gel H_3_[PW_12_O_40_] (2016)	0.7^b^	-	MW	130 °C	15^e^	78.9
	2	HPA (2013)	5^b^	-	MW	-	10^e^	95
	3	K_7_Na_3_P_2_W_18_-Cu_4_O_68_ (2012)	0.2^b^	-	Heat	140 °C	90^e^	92
	4	PWA-SiO_2_ (2011)	0.2^a^	-	Heat	100 °C	8^e^	98
	5	H_14_[NaP_5_W_30_O_110_] (2008)	0.01^b^	Acetonitrile	Heat	82 °C	0.5^h^	95
	6	WD-SiO_2_ (2009)	0.3^a^	-	Heat	140 °C	2^d^	85
				-	MW	-	10^e^	90
	7	H_4_[PMo_11_VO_40_] (2006)	1^b^	Ethanol	Heat	78 °C	5^e^	84
**PPA**	8	PPA-SiO2 (2011)	0.01^a^	-	MW	-	15^e^	96
**Alumina**	9	ZrO_2-_Al_2_O_3_ (2016)	0.03^a^	-	Heat	120 °C	20^e^	99.2
	10	Acidic Al_2_O_3_-NH_4_OAc (2000)	2.5^a^	-	MW	-	20^e^	82

(Table 4 summarizes heteropolyacid and ions, PPA and alumina based different catalysts and covers the reaction conditions, catalyst amount and yields under which variety of catalysts investigated. Table 4 emphasized the information of individual catalyst only, comparison between catalysts cannot be considered due to diversely substituted reactants.)

**Table 5:** Magnetic nanocatalysts for synthesis of imidazole derivatives.

Types of solid or polymer nano catalysts	Entry	Polymer support catalysts	Quantity of catalyst (^a^g,^b^mol %,^c^ wt %)	Solvent Conditi-ons	Energy medium	Temp. in °C . (rt = room temp.)	Time (^d^ h, ^e^ min, ^f^ s)	Yield (%)
**Magnetic nano catalysts**	1	Fe_3_O_4_@HA (2021)	0.01^c^	Water	Heat	Rt	3^e^	99
	2	Cu_2_O/Fe_3_O_4_ @guarana (2021)	0.01^c^	Ethanol	Ultrasonic	Rt	20^e^	97
	3	HMS-SA (2020)	0.03^a^	Ethanol	Ultrasonic	-	25^e^	99
	4	ZnS-ZnFe_2_O_4_ (2019)	0.02^a^	Ethanol	Ultrasonic	70 °C	15^e^	95
	5	Cu@imine/Fe_3_O_4_ (2019)	0.36^b^	-	Heat	80 °C	35^e^	95
	6	Fe_3_O_4_/SO_3_H@ zeolite-Y (2019)	0.02^c^	Ethanol	Heat	80 °C	45^e^	98
	7	ZnS/CuFe_2_O_4_ (2019)	0.03^a^	Ethanol	Heat	80 °C	-	92
	8	(Fe_3_O_4_@SiO_2_@Si (CH_2_)_3_@N-Ligand@Co) (2019)	0.05^a^	-	Heat	100 °C	25^e^	90
	9	Fe_3_O_4_@PVA-SO_3_H (2019)	0.04^a^	Ethanol	Heat	Rt	40^e^	95
	10	Fe_3_O_4_@SiO_2_/BiPy^+2^ 2Cl^−^ (2019)	0.05^a^	-	Heat	120 °C	20^e^	90
	11	Fe_3_O_4_@Ca_3_(PO_4_)_2_ (2018)	0.05^a^	-	Heat	95 °C	46^e^	90
	12	[P_4_-VP]-Fe_3_O_4_ (2018)	0.1^a^	-	Heat	100 °C	35^e^	98
	13	Fe_3_O_4_@SiO_2_@ (CH_2_*)*_3_N^+^Me_3_I_3_^−^ (2017)	0.007^a^	-	Heat	120 °C	15^e^	94
	14	NZF@HA-PRS (2017)	0.02^a^	-	Heat	Rt	30^e^	91
	15	Fe_3_O_4_@_g_-C_3_N_4_ (2017)	0.2^a^	Ethanol	Heat	78 °C	2^d^	95
	16	n-Fe_3_O_4_@ZrO_2_ /PMA (2017)	0.0025^a^	-	Heat	110 °C	8^e^	98
	17	γ-Fe_2_O_3_@ TiO_2_-EG- Cu(II) (2017)	0.030^a^	-	Heat	100 °C	20^e^	98
	18	ZnFe_2_O_4_ 2017)	0.005^b^	-	Heat	80 °C	30^e^	96
	19	Fe_3_O_4_@ SiO_2_@ PolyIonene/Br_3_^−^ (2016)	0.05^a^	-	Heat	110 °C	15^e^	91
	20	Nano-CuFe_2_O_4_ (2010)	0.002^b^	Ethanol	Ultrasound	Rt	30^e^	96
	21	Cu/GA/Fe_3_O_4_ @SiO_2_ (2016)	0.010^a^	-	Heat	90 °C	60^e^	92
	22	NiFe_2_O_4_@ SiO_2_H_3_PMo_12_O_40_ (2015)	0.02^a^	-	Heat	120 °C	20^e^	94
	23	Fe3O4–PEG–Cu (2014)	10^b^	-	Heat	110 °C	30^e^	98

(Table 5 summarizes different magnetic nanocatalysts and covers the reaction conditions, catalyst amount and yields under which variety of catalysts investigated. Table 5 emphasized the information of individual catalyst only, comparison between catalysts cannot be considered due to diversely substituted reactants.)

**Table 6:** Silica based nanocatalysts for synthesis of imidazole derivatives.

Types of solid or polymer nanocatalysts	Entry	Polymer support catalysts	Quantity of catalyst (^a^g,^b^mol %,^c^ wt %)	Solvent conditions	Energy medium	Temp. in °C . (rt = room temp.)	Time (^d^ h, ^e^ min, ^f^ s)	Yield (%)
**Silica based nano catalysts**	1	BNPs@ SiO_2_- TPPTSA [2020]	0.05^a^	-	Heat	140 °C	38^e^	97
	2	SiSAIn (OTf)_2_ (2019)	0.05^a^	Ethanol: water	Heat	80 °C	10^e^	80
	3	Nano-SiO_2_ (2017)	5^b^	Ethanol	Heat	80 °C	15^e^	97
	4	Dendrimer- PWA^n^ (2016)	0.035^a^	-	Heat	90 °C	30^e^	96
			0.04^a^	Ethanol	Ultrasound	rt	15^e^	94
	5	PMO-ICS (2016)	0.02^a^	Ethanol	Heat	Reflux	42^e^	98
	6	HNO_3_@ nano SiO_2_ (2015)	0.012^a^	-	Heat	100 °C	3.45^d^	91
	7	NHSO_3_H-KIT-5 (2015)	0.05^a^	-	Heat	120 °C	10^e^	90
	8	FHS/SiO_2_ (2015)	0.04^e^	-	Heat	110 °C	30^e^	95
	9	Mo(IV) salen complex (2014)	2.5^b^	Ethanol	Heat	50 °C	1^d^	94
	10	Silica-Supported Preyssler (2013)	0.03^a^	-	Heat	Reflux	3^d^	98.5
	11	Nano-SnCl_4_ / SiO_2_ (2013)	0.03^a^	-	Heat	130 °C	2^d^	96
	12	Nano-SPA (2012/13)	0.04^a^	-	Heat	140 °C	3^d^	92
			0.06^a^	-	Heat	140 °C	2^d^	94
	13	PSNP-CA (2013)	0.01^a^	Water	Heat	140 °C	4^d^	94
	14	Nano-TiCl_4_- SiO_2_ (2011)	0.2^a^	-	Heat	110 °C	0.5^d^	95

(Table 6 summarizes numerous silica based nanocatalysts and covers the reaction conditions, catalyst amount and yields under which variety of catalysts investigated. Table 5 emphasized the information of individual catalyst only, comparison between catalysts cannot be considered due to diversely substituted reactants.)

**Table 7:** Biohybrid and metal oxide nanocatalysts for substituted imidazole synthesis.

Types of solid or polymer nano catalysts	Entry	Polymer support catalysts	Quantity of catalyst (^a^g,^b^mol %,^c^ wt %)	Solvent conditions	Energy medium	Temp. in °C . (rt = room temp.)	Time (^d^ h, ^e^ min, ^f^ s)	Yield (%)
**Biohybrid nanocatalysts**	1	Ag NP-CS (2018)	0.04^a^	-	Heat	100 °C	100^e^	90
	2	CS NPs/MWCNT @Fe_3_O_4_ (2017)	0.020^a^	Ethanol	Heat	Reflux	60^e^	95
	3	MIP-NPs (2017)	0.04^a^	-	Heat	110 °C	40^e^	97
	4	MCS-GT@ Co(II) (2017)	0.005^a^	Ethanol	Heat	Reflux	39^e^	95
	5	cellulose/g- Fe_2_O_3/_Ag (2016)	0.015^a^	-	Heat	100 °C	10^e^	91
	6	n-TiO_2_@TDI @chitosan (2016)	0.02^a^	-	Heat	100 °C	30^e^	93
	7	GO–Chitosan (2015)	0.012^a^	-	Heat	120 °C	10^e^	95
	8	ZrO_2_-β-CD (2015)	40^b^	Water	Heat	100 °C	0.4^h^	96
	9	Fe_3_O_4_@ CS (2014)	0.05^a^	Ethanol	Heat	Reflux	2^h^	95
**Metal oxide nano catalysts**	10	Au@ RGO (2021)	0.054^c^	Water	Heat	50 °C	6^d^	90
	11	Plant assisted tin oxide (2020)	0.5^a^	-	MW	-	180^f^	96
	12	rGO-NiO-NCs (2020)	0.025^a^	Ethanol	Heat	55 °C	60^e^	96
	13	ZnO-NPs (2020)	10^b^	Methanol	Heat	Reflux	1^d^	82
				-	MW	160 °C	15^e^	90
	14	ZnO-NPs (2019)	0.1^b^	Glacial acetic acid	MW	60 °C	2^d^	82
	15	S-LPMO (2018)	0.04^a^	Ethanol	Heat	Reflux	2^d^	94
	16	Ag–Fe/ZSM-5 (2016)	5^c^	-	Heat	120 °C	1^d^	95
	17	TiO_2_@SiO_2_ (2014)	0.04^a^	Methanol	Heat	Rt	2^d^	90
	18	Fe–Cu/ZSM-5 (2014)	3^c^	-	Heat	70 °C	70^e^	99
	19	SiO_2_-SnO_2_ (2012)	0.5^b^	-	Heat	80 °C	25^e^	94

(Table 7 summarizes variety of biohybrid and metal oxide based nanocatalysts which covers the reaction conditions, catalyst amount and yields under which variety of catalysts investigated. Table 7 emphasized the information of individual catalyst only, comparison between catalysts cannot be considered due to diversely substituted reactants.)

**Table 8:** Clay and minerals and Ionic liquid based with miscellaneous nanocatalysts for synthesis imidazole derivatives.

Types of solid or polymer nanocatalysts	Entry	Polymer support catalysts	Quantity of catalyst (^a^g,^b^mol %,^c^ wt %)	Solvent conditions	Energy medium	Temp. in °C . (rt = room temp.)	Time (^d^ h, ^e^ min, ^f^ s)	Yield (%)
**Clay and mineral nano catalysts**	1	Nano kaolin-SO_3_H (2018)	0.05^a^	-	Heat	140 °C	7^e^	95
	2	n-CTW-SA (2016)	0.006^a^	-	Heat	100 °C	10^e^	99
	3	n-PeFBA (2015)	0.01^b^	-	Heat	100 °C	35^e^	98
	4	Cu/SAPO-34 (2015)	5^c^	Water	Ultrasound	Rt	5^e^	95
**Ionic liquid nano catalysts**	5	[H-NP]HSO_4_ (2018)	0.016^a^	-	Heat	90 °C	20^e^	95
	6	H_3_PW_12_O_40_- Fe_3_O_4_@ SiO_2_ – Pr–Pi (2018)	0.02^a^	Ethanol	Heat	Reflux	1^d^	95
	7	Fe_3_O_4_@SiO_2_. HM.SO_3_H (2015)	0.03^a^	-	MW	-	7^e^	91
	8	MNPs-IL (2013)	0.1^a^	Ethanol	Ultrasound	Rt	30^e^	94
			0.1^a^	-	Heat	130 °C	30^e^	90
			0.1^a^	-	MW	-	15^e^	95
**Miscellaneous nano catalysts**	9	Cu/C (2019)	1^b^	PEG -200	Heat	100 °C	1.5^d^	93
	10	Xantphos (Ru4 NPs) (2017)	1^b^	DMF	Heat	85 °C	1^d^	89
	11	n-TiO_2_–NH_2_/HPW (2017)	0.02^a^	-	Heat	97 °C	42^e^	95.5

(Table 8 summarizes numerous clay and mineral based nanocatalysts, ionic liquid nanocatalysts and miscellaneous nanocatalysts based catalysts and covers the reaction conditions, catalyst amount and yields under which variety of catalysts investigated. Table 8 emphasized the information of individual catalyst only, comparison between catalysts cannot be considered due to diversely substituted reactants.)

**Table 9:** Organic polymers based catalysts for synthesis imidazole derivatives.

Types of organic solid or polymer catalysts	Entry	Polymer support catalysts	Quantity of catalyst (^a^g,^b^mol %,^c^ wt %)	Solvent conditions	Energy medium	Temp. in °C . (rt = room temp.)	Time (^d^ h, ^e^ min, ^f^ s)	Yield (%)
**Polymers and copolymers**	1	SMI-SO_3_H (2014)	0.08 ^a^	-	Heat	100 °C	10^e^	93
	2	Poly (AMPS-co-AA) (2011–12)	0.03^a^	-	Heat	110 °C	25^e^	95
			0.03^a^	-	Heat	110 °C	25^e^	90
**Resins**	3	Amberlite IR 120H^+^ (2017)	10^c^	Ethanol	Heat	Reflux	10^e^	93
	4	Amberlyst A-15 (2011)	0.35^a^	-	MW	-	12^e^	92
**PEG**	5	PEG-400 (2016)	-	PEG-400	Heat	rt	4^d^	91
	6	PTC (PEG400) (2014)	10^b^	Glacial acetic acid	Heat	Reflux	5^d^	97
	7	- (2010)	-	PEG-600	MW	-	5^e^	80
**Polymeric Carbon**	8	Carbon-based solid acids (2010)	0.2^a^	-	Heat	130 °C	2^d^	90

(Table 9 summarizes organic polymeric catalysts of different categories; polymers and copolymers, resins, PEG and polymeric carbon as catalysts which covers the reaction conditions, catalyst amount and yields under which variety of catalysts investigated. Table 9 emphasized the information of individual catalyst only, comparison between catalysts cannot be considered due to diversely substituted reactants.)

**Table 10:** Hybrid polymers based catalysts for substituted imidazole synthesis.

Types of hybrid solid or polymer catalysts	Entry	Polymer support catalysts	Quantity of catalyst (^a^g,^b^mol %,^c^ wt %)	Solvent conditions	Energy medium	Temp. in °C . (rt = room temp.)	Time (^d^ h, ^e^ min, ^f^ s)	Yield (%)
**Organo silica**	1	SBA-15– Pr–SO_3_H (2018)	3^c^	-	MW	-	3^e^	99
	2	PS@SiTPA30 (2016)	1^b^	-	Heat	130 °C	90	93
	3	SiO_2_-Pr- SO_3_H (2013)	0.02^b^	-	Heat	140 °C	10^e^	98
**Metal- organic framework (MOF)**	4	MIL-101(Cr)- MOF (2021)	0.005^a^	-	Heat	120 °C	10^e^	95
	5	(Co/Ni)- MOF (2016)	0.015^a^	-	Heat	120 °C	10^e^	95
**Miscellaneous**	6	Cu (I) Modified- SBA-15 (2017)	0.01^a^	-	Heat	100 °C	60^e^	98
	7	Polymer- supported ZnCl_2_ (2008)	20^b^	Ethanol	Heat	78 °C	1.5^d^	97

(Table 10 summarizes hybrid polymeric catalysts of different categories like organosilica and MOF and also miscellaneous polymeric catalysts which covers the reaction conditions, catalyst amount and yields under which variety of catalysts investigated. Table 10 emphasized the information of individual catalyst only, comparison between catalysts cannot be considered due to diversely substituted reactants.)

**Table 11:** Polymer based biocatalysts for synthesis of imidazole derivatives.

Types of solid or polymer biocatalysts	Entry	Polymer support catalysts	Quantity of catalyst (^a^g,^b^mol %,^c^ wt %)	Solvent conditions	Energy medium	Temp. in °C . (rt = room temp.)	Time (^d^ h, ^e^ min, ^f^ s)	Yield (%)
**Biocatalysts**	1	Baker’s yeast (2021)	2^a^	Methanol	Stirring	rt	4.5^d^	92
	2	Cellulose@ pumice (2020)	0.03^a^	Ethanol	Stirring	25 °C	25^e^	97
	3	OAc-HPro@ Fe_3_O_4_ (2017)	0.04^a^	Ethanol	Heat	60 °C	7^d^	99
	4	COPAPSC (2015)	0.2^a^	-	Heat	110 °C	4^d^	84
	5	Chitosan- SO_3_H (2015)	0.1^a^	Ethanol	MW	100 °C	7^e^	91
	6	Lipase (2012)	0.015^a^	Ethanol	Heat	45 °C	9^d^	75
	7	Bio Glycerol- based carbon catalyst (2011)	10^c^	CH_3_CN	Heat	50 °C	7^d^	84
	8	Cellulose-SO_3_H (2010)	0.1^a^	-	MW	-	2^e^	98
	9	La-CMC (2009)	0.02^a^	THF	Heat	Reflux	3^d^	93

(Table 11 summarizes polymeric biocatalyst and covers the reaction conditions, catalyst amount and yields under which variety of catalysts investigated. Table 11 emphasized the information of individual catalyst only, comparison between catalysts cannot be considered due to diversely substituted reactants.)
